# Synthesis and luminescence properties of substituted benzils

**DOI:** 10.1038/s42004-023-01038-6

**Published:** 2023-11-09

**Authors:** Masamichi Yasui, Takashi Fujihara, Hiroyoshi Ohtsu, Yuki Wada, Terumasa Shimada, Yiying Zhu, Masaki Kawano, Kengo Hanaya, Takeshi Sugai, Shuhei Higashibayashi

**Affiliations:** 1 Faculty of Pharmacy, Keio University, 1-5-30 Shibakoen, Minato-ku, Tokyo 105-8512 Japan; 2 Department of Chemistry, Graduate School of Science, Chiba University, 1-33 Yayoi, Inage, Chiba, 263-8522 Japan; 3 Comprehensive Analysis Center for Science, Saitama University, Shimo-okubo, Sakura-ku, Saitama-city, Saitama, 338-8570 Japan; 4 Department of Chemistry, School of Science, Tokyo Institute of Technology, 2-12-1 Ookayama, Meguro-ku, Tokyo 152-8550 Japan

**Keywords:** Organic chemistry, Chemical synthesis, Photochemistry, Synthetic chemistry methodology

## Abstract

Photophysical properties of benzil (1,2-diphenylethane-1,2-dione) and its derivatives in the crystal state have recently attracted much attention. However, the study of substituted benzils has mostly been limited to *para*-substituted derivatives, which did not induce a significant effect on the emission wavelength compared to pristine benzil. The effects of *ortho*- and *meta*-substituents on the photophysical properties in the crystal state have not been investigated so far. Our recently developed organocatalytic pinacol coupling of substituted benzaldehydes allowed us to prepare various *ortho*-, *meta*-, and *para*-substituted benzil derivatives and to investigate their luminescence properties. *Ortho*- and *meta*-substituents affected the electronic states of benzils in the crystal state, resulting in differences in their luminescence properties. The luminescence wavelength and type, i.e., phosphorescence or fluorescence, were altered by these substituents. Fast self-recovering phosphorescence-to-phosphorescence mechanochromism by the *para*-CF_3_ substituent at room temperature was also discovered.

## Abstract (web summary)

Benzil and its derivatives have been widely studied for their luminescence properties, but the effects of *ortho*- and *meta*-substituents on photophysical properties have yet to be explored. Here, *ortho*-, *meta*- and *para*-substituted benzil derivatives are accessed via an organocatalytic pinacol coupling of substituted benzaldehydes, and *ortho*- and *meta*-substituents are shown to significantly influence the electronic state and luminescence properties of benzil.

## Introduction

Photophysical properties of benzil (1,2-diphenylethane-1,2-dione) and its derivatives have attracted much attention. In particular, phosphorescence, fluorescence, and photorotamerization in solution, frozen matrices, or in host molecules have been extensively studied^1–9^. More recent research has focused on the modification of photophysical properties in crystal states of substituted benzil derivatives^10–17^. Tang’s group studied the luminescence properties of crystals of benzil and its *para*-substituted F, Br, CH_3_, and OCH_3_ derivatives^11^. Although these benzils were emissive in the crystal state, the *para*-substituents did not induce a significant effect on the emission wavelength (500–526 nm) compared with that of pristine benzil (521 nm). *para*-Carbazolyl groups, *para*-bromine atoms, and *para*-alkoxyl groups have induced polymorphism-dependent phosphorescence^12^, formed phosphorescent crystals with elastic and plastic bending properties^13^, and generated stimuli-responsive phosphorescence^17^, respectively. In contrast to these studies on *para*-substituents, only one study has been reported for *ortho*-substituents in the crystal state. Tani’s group reported that *ortho*-halogens cause phosphorescence mechanochromism on a 2,2’-thenil [1,2-di(2-thiophenyl)ethane-1,2-dione] skeleton with chalcogen bonding between carbonyl groups and thiophene moieties^14–16^. The effects of *ortho*- and *meta*-substituents of the benzil skeleton on the photophysical properties in the crystal state have not been investigated so far. This may be due to the steric difficulties in preparing *ortho*-substituted derivatives. Recently, we developed an organocatalytic pinacol coupling of substituted benzaldehydes^18^, which allowed us to prepare various *ortho*-, *meta*-, and *para*-substituted benzil derivatives and to investigate their luminescence properties.

**Fig. 1:**
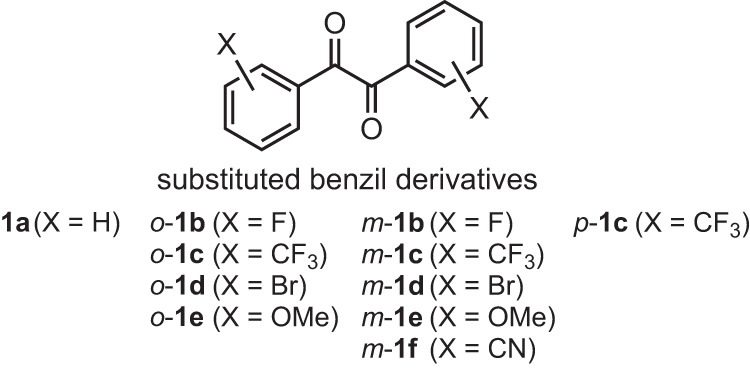
*ortho*-, *meta*-, and *para*-Substituted benzil derivatives. Substituted benzil derivatives whose luminescence properties were studied in this work.

In this study, we revealed that the electronic state of benzil was significantly affected by *ortho*- and *meta*-substituents, creating differences in their luminescence properties in the crystal state. The emission wavelengths of the crystals were blue-shifted or red-shifted by the effect of *ortho*-substituents in the crystal state, unlike reported *para*-substituents^11^, and the types of the luminescence (i.e., fluorescence or phosphorescence) in the crystal state were altered by the position and type of substituents. In addition, a *para*-substituted benzil derivative with CF_3_ was found to exhibit fast self-recovering phosphorescence-to-phosphorescence mechanochromism at room temperature. Here, we report the details of the luminescence properties of benzils altered by *ortho*- and *meta*-substituents and the mechanochromic phosphorescence induced by *para*-CF_3_ (Fig. 1).

## Results and discussion

**Fig. 2:**
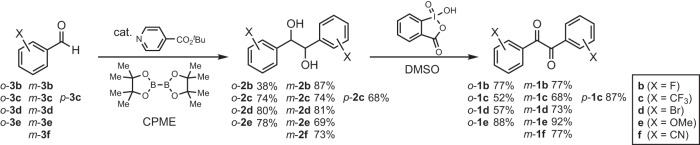
Synthesis of substituted benzils 1. CPME cyclopentyl methyl ether, DMSO dimethyl sulfoxide.

**Fig. 3:**
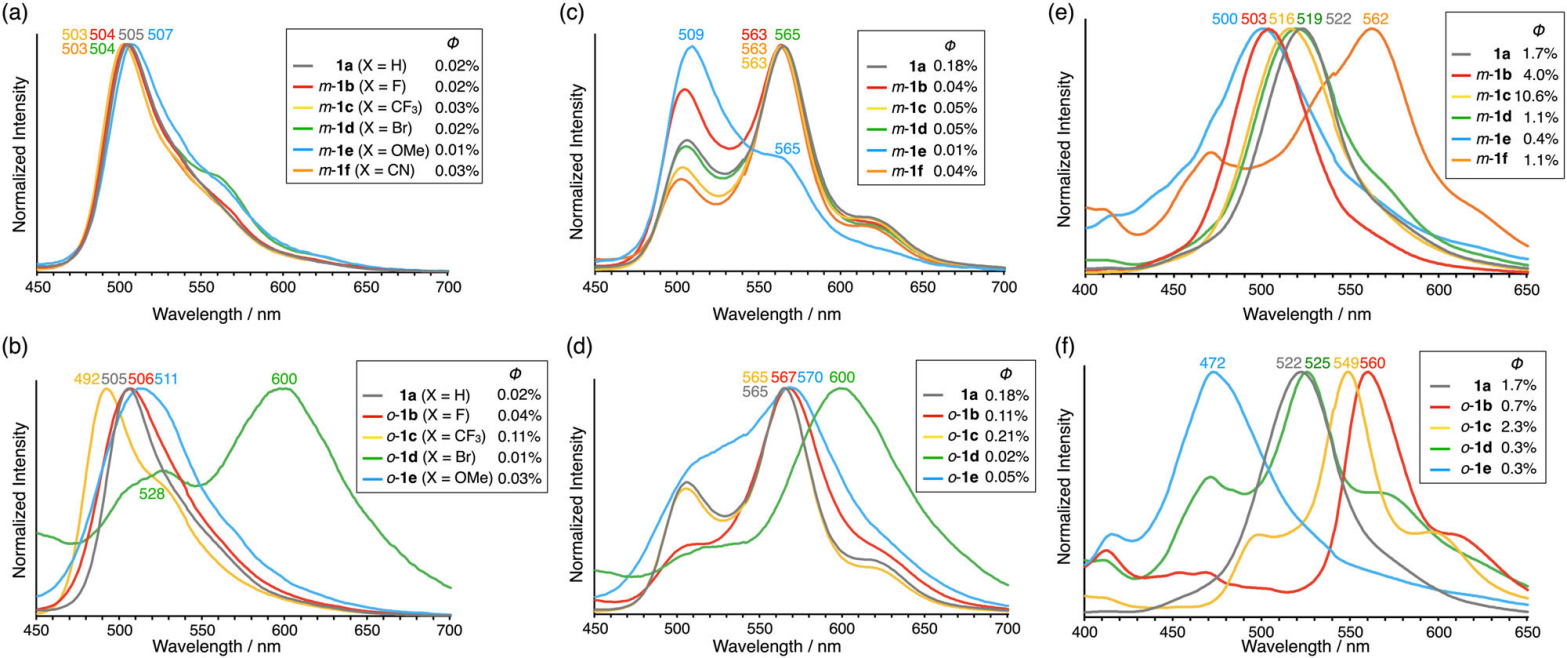
Emission spectra of *m*-1 and *o*-1. Emission spectra and quantum yields **a**, **b** under air, **c**, **d** under Ar in cyclohexane (5.0 × 10^-5^ M, excited at 260 nm), and **e**, **f** in crystal state (excited at 260 nm).

**Fig. 4:**
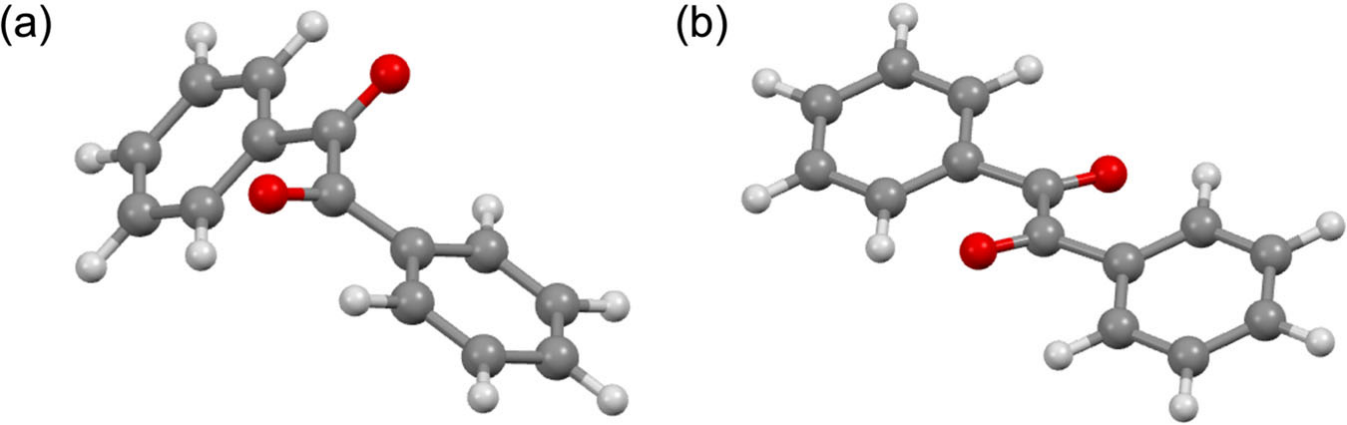
Conformers of benzil. **a** *cis*-Skew and **b** *trans*-planar conformers.

Substituted benzil derivatives *o*-**1b**-**e,** *m*-**1b**-**f** with F, CF_3_, Br, MeO, CN groups and *p*-**1c** with CF_3_ were synthesized from substituted benzaldehydes **3** according to our reported pinacol coupling^18^ followed by oxidation of diols **2** by 2-iodoxybenzoic acid (Fig. 2, Methods, and Supplementary Information). The photophysical properties of *o*-**1b**-**e** and *m*-**1b**-**f** in solution were first investigated. The UV-vis absorption spectra and emission spectra under air and Ar in cyclohexane were measured (Supplementary Fig. 1, Fig. 3a–d). UV-vis absorption spectra of *m*-**1b**-**f** exhibited similar spectra to the parent **1a** (Supplementary Fig. 1a). The band at 319 nm of *m*-**1e** (X = OMe) was assigned to the π-π* transition of the benzoyl moiety by TD-DFT calculations [cam-B3LYP/6-311 + G(d,p)//B3LYP/6-311 + G(d,p)]. In the UV-vis absorption spectra of *o*-**1b**-**e**, more perturbation on the wavelength and absorbance by *o*-substituents was observed than for the *m*-substituents (Supplementary Fig. 1b). Emission spectra under air in Fig. 3a, b and under Ar in Fig. 3c, d show fluorescence around 500 nm and phosphorescence around 560 nm. The emission wavelengths were similar in both *m*-**1** and *o*-**1**. The phosphorescence of only *m*-**1e** (X = OMe) was weak even under Ar. *o*-**1d** with Br exhibited phosphorescence even under air, with an emission wavelength slightly red-shifted from the others, which had already been reported by Tani’s group^14^. The luminescence property of benzils in solution through photorotamerization from the *cis*-skew conformer in the ground state to the *trans*-planar conformer (Fig. 4) has been well studied^1–9^.

Next, we investigated the luminescence properties of *m*-**1b**-**f** and *o*-**1b**-**e** in the crystal state. Emission spectra of crystals of *m*-**1b**-**f** and *o*-**1b**-**e**, obtained from EtOH/H_2_O, are shown in Fig. 3e, f. *m*-**1b**-**e** exhibited similar emission maximum wavelengths (500–519 nm) to parent **1a** (522 nm). The emission maximum of *m*-**1f** (X = CN) was exceptionally red-shifted to 562 nm. It is notable that the quantum yields (*Φ*) of *m*-**1b** (X = F) and especially *m*-**1c** (X = CF_3_) were enhanced by the introduction of F and CF_3_ groups to 4.0 and 10.6%, respectively. In contrast to *m*-**1**, the emission maxima of *o*-**1** were found to be greatly affected by the *o*-substituents. The emission wavelengths of *o*-**1b** (X = F) and *o*-**1c** (X = CF_3_) with electron-drawing groups were red-shifted to 560 and 549 nm, respectively, and that of *o*-**1e** (X = OMe) with an electron-donating group was blue-shifted to 472 nm.

From comparing the luminescence of **1a**-**f** between the solution and crystal states, it was anticipated that the difference in emission wavelengths could be derived from the type of luminescence, i.e., fluorescence for short wavelengths and phosphorescence for long wavelengths. Only luminescence lifetimes of **1a,** *para*-substituted benzils with carbazolyl, Br, or alkoxy groups^12,13,17^ and 2,2’-thenils have been reported, while those of benzils with various substituents have not been systematically investigated. To clarify the difference in the emission wavelengths of substituted benzils **1** in the crystal state, we measured the luminescence lifetimes of *m*-**1b**-**f** and *o*-**1b**-**e** (Table 1, Supplementary Fig. 2). While the types of the luminescence were found to be altered by the position and type of substituents, the results were not as expected. *m*-**1b**-**f** with similar emission maxima (except *m*-**1f**) exhibited phosphorescence or fluorescence depending on the substituents, regardless of the emission wavelengths. In contrast to the phosphorescent nature of parent **1a**^11^, the luminescence lifetimes of *m*-**1d** (X = Br), *m*-**1e** (X = OMe), and *m*-**1f** (X = CN) with emissions at 519, 500, 562 nm were 1.73, 1.41, and 1.68 ns, indicating that their short-lived emissions are fluorescence. On the other hand, the lifetime of *m*-**1b** (X = F) with emission at 503 nm was 302 μs, which is assigned to phosphorescence. *m*-**1c** (X = CF_3_) with emission at 516 nm exhibited both prompt (1.13 ns) and delayed (1.75 ms) luminescence. The temperature-dependent emission spectra of *m*-**1c** at 293 to 333 K showed a gradual decrease in the emission intensity with increasing temperature (Supplementary Fig. 3), indicating that the delayed luminescence is phosphorescence and not thermally activated delayed fluorescence (TADF). In contrast, the luminescence of *o*-**1b**-**e** showing diverse emission maxima at 472-560 nm was found to be all fluorescence with short lifetimes (1.22–3.43 ns).

**Table 1:** Emission wavelength, lifetime, emission type in crystal state, calculated^a^ conformation, S_1_, T_3_, and energy gap Δ*E*_S-T_ between S_1_ and T_3_.

1	Wavelength/nm	Lifetime	Emission type^b^	Conformation	S_1_/eV	T_3_/eV	Δ*E*_S-T_/eV
**1a**	522	142 μs^11^	*P*	*cis*-skew	3.21	3.13	0.08
*m*-**1b** (X = F)	503	302 μs	*P*	*cis*-skew	3.16	3.08	0.08
*m*-**1c (**X = CF_3_)	516	1.75 ms, 1.13 ns	*P*, *F*	*cis*-skew	3.15	3.12	0.03
*m*-**1d** (X = Br)	519	1.73 ns	*F*	*cis*-skew	3.18	3.05	0.13
*m*-**1e** (X = OMe)	500	1.41 ns	*F*	*cis*-skew	3.26	3.07	0.19
*m*-**1f** (X = CN)	562	1.68 ns	*F*	*cis*-skew	3.12	3.02	0.10
*o*-**1b** (X = F)	560	2.47 ns	*F*	*cis*-skew	3.47	3.23	0.24
*o*-**1c** (X = CF_3_)	549	3.43 ns	*F*	*trans*-planar	2.79	-	-
*o* -**1d** (X = Br)	525	1.95 ns	*F*	*cis*-skew	3.37	3.17	0.20
*o*-**1e** (X = OMe)	472	1.22 ns	*F*	*cis*-skew	3.56	3.17	0.39

^a^cam-B3LYP/6-311 + G(d,p)// B3LYP/6-311 + G(d,p).

^b^*P* phosphorescence, *F* fluorescence.

**Fig. 5:**
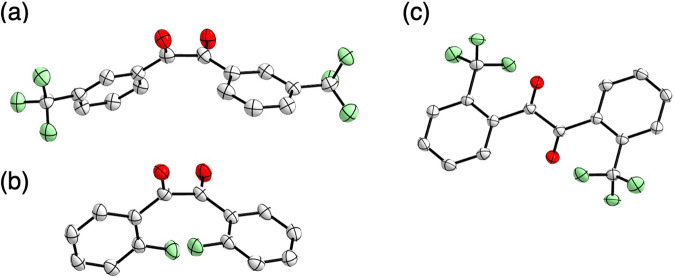
ORTEP drawings of *m*-1c, *o*-1b, and *o*-1c by X-ray analysis. **a** *m*-**1c**, **b** *o*-**1b**, and **c** *o*-**1c** at the 50% probability level. Hydrogens are omitted for clarity.

To understand the unexpected different emission properties of *m*-**1b**-**f** and *o*-**1b**-**e** by the position and type of substituents, DFT calculations were performed. Since benzil possesses *cis*-skew and *trans*-planar conformers, the most stable conformers of **1a,** *m*-**1b**-**f**, and *o*-**1b**-**e** in the ground state were determined by DFT calculations [B3LYP/6-311 + G(d,p)]. The most stable conformers were *cis*-skew for all compounds except for *o*-**1c** (X = CF_3_) which was *trans*-planar (Table 1, Supplementary Figs. 4, 5). The X-ray structures of **1a** and *o*-**1d** (X = Br) were previously found to be in the *cis*-skew conformation in the crystal state^19,20^. X-ray analyses of single crystals of *m*-**1c** (X = CF_3_), *o*-**1b** (X = F), and *o*-**1c** (X = CF_3_) elucidated the *cis*-skew structures of *m*-**1c,** *o*-**1b** and the *trans*-planar structure of *o*-**1c** in the crystal state (Fig. 5, Supplementary Data 3–6). The calculated stable conformations were consistent with these experimental structures. It was reported that *cis*-skew conformer is most stable conformation in the ground state of benzil^21–24^. Since it was reported that bulky alkyl groups at the *ortho*-positions caused *trans*-planar conformer^21^, stable *trans*-planar conformer of *o*-**1c** is also attributed to the steric hindrance of CF_3_ groups. Although other single crystals were not obtained, we assumed that they also possess the calculated *cis*-skew conformation in the crystal state. While **1a** shows the photorotamerization in the solution^1^, the conformation in the crystal state is not changed. The difference between the luminescence in the solution and that in the crystal state was primarily explained by the difference of the conformational dynamics without significant intermolecular interactions as reported^10,11^. We also analyzed the excited states of *m*-**1b**-**f** and *o*-**1b**-**e** based on the calculated stable structures of single molecules.

The energies of excited states S_1_ and T_1_-T_3_ of **1a,** *m*-**1b**-**f**, and *o*-**1b**-**e** were calculated by the TD-DFT method [cam-B3LYP/6-311 + G(d,p)] (Table 1, Supplementary Table 1, Supplementary Figs. 6, 7). The closest triplet excited state to the S_1_ state was the T_3_ state in **1a,** *m*-**1b**-**f**, and *o*-**1b,d,e** with *cis*-skew conformations (Table 1, Supplementary Table 1). According to the El-Sayed rule, a transition from ^1^(n, π*) to ^3^(π, π*) is allowed due to its large spin-orbit coupling (SOC) value in contrast to a forbidden transition from ^1^(n, π*) to ^3^(n, π*)^5^. Since S_1_ and T_3_ of benzils **1** each have (n, π*) and (π, π*) characteristics and the S_1_/T_3_ SOC values for **1a,** *m*-**1b**-**f**, and *o*-**1b,d,e** calculated by the TD-DFT method were large (Supplementary Figs. 6, 7), the intersystem crossing (ISC) from S_1_ to T_3_ is allowed in the El-Sayed rule. ISC also depends on the energy gap Δ*E*_S-T_ between the S and T states. The energy gaps Δ*E*_S-T_ between S_1_ and T_3_ in the calculation of benzils **1a,** *m*-**1b**-**f**, and *o*-**1b,d,e** varied between 0.03–0.39 eV depending on the position and type of substituents (Table 1). Judging from the observed emission type and the calculated values of Δ*E*_S-T_, **1a** and *m*-**1b,c** with *E*_S-T_ smaller than 0.08 eV undergo ISC, thus exhibiting phosphorescence. On the other hand, the Δ*E*_S-T_ of *m*-**1d**-**f** and *o*-**1b,d,e** larger than 0.10 eV do not allow ISC, resulting in fluorescence. In *o*-**1c**, the closest triplet excited state to the S_1_ state was the T_1_ state (Supplementary Table 1). Since the S_1_/T_1_ SOC with ^1^(n, π*) and ^3^(n, π*) characteristics was 0 cm^-1^ (Supplementary Fig. 7), the ISC from S_1_ to T_1_ is forbidden, thus *o*-**1c** shows fluorescence.

The difference in fluorescence wavelengths of *o*-**1** can be attributed to the *o*-substituent effect on the energy of the S_1_ state except for *o*-**1b** (X = F)^25–27^. TD-DFT calculations showed that the energy of the S_1_ states becomes higher in the order of *o*-**1c,** *o*-**1d**, and *o*-**1e** (Table 1), which agreed with that of the observed fluorescence wavelengths. The long-wavelength fluorescence of *m*-**1f** (X = CN) is also attributed to the low energy of the S1 state (Table 1). The relatively high energy of the S_1_ state calculated for *o*-**1b** is not consistent with the order of experimentally observed wavelengths. Since the calculation is based on the structure of single molecule, the discrepancy of *o*-**1b** is attributed to intermolecular interactions in the crystal state. However, the exact intermolecular interaction causing the longer fluorescence wavelength is not clear.

**Fig. 6:**
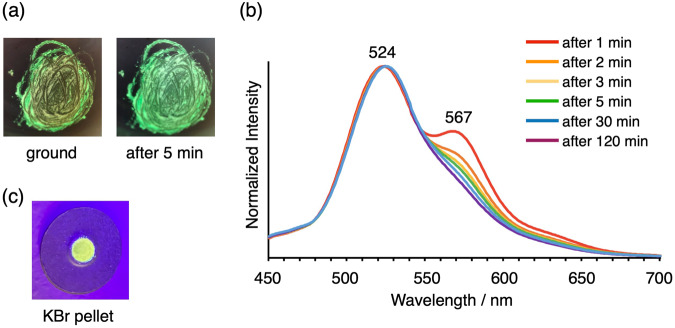
Fast self-recovering phosphorescence-to-phosphorescence mechanochromism of *p*-1c at room temperature. **a** Images of the powder of *p*-**1c** just after grinding and after 5 min under irradiation at 254 nm. **b** Emission spectral change of *p*-**1c** after grinding (excited at 260 nm). **c** Image of KBr plate of *p*-**1c** under irradiation at 365 nm.

Finally, the higher quantum yields of *m*-**1c** and *o*-**1c** (X = CF_3_) found in this study motivated us to investigate the luminescence property of *para*-substituted **1c** (X = CF_3_). Notably, *p*-**1c** was found to exhibit fast self-recovering phosphorescence-to-phosphorescence mechanochromism at room temperature. Phosphorescent mechanochromism of benzil derivatives was reported by both Tani’s and Ma’s groups in a substituted 2,2’-thenil and a *p*-alkoxybenzil as phosphorescence in the crystal state and phosphorescence in the amorphous state, respectively^14,17^. *p*-**1c** showed green phosphorescence at 524 nm with *Φ* = 7.5% and lifetime = 1.86 ms (Supplementary Fig. 8). Grinding the powder changed the green phosphorescence to yellow luminescence, and the green phosphorescence was recovered in a few minutes at room temperature (Fig. 6a). The emission spectra of the ground power showed a new emission band at 567 nm, which quickly decreased in a few minutes in the time-dependent spectral measurements (Fig. 6b). Most organic mechanochromic luminescent compounds need exposure to solvents or heating to recover their pristine luminescence. Fast self-recovering mechanochromic luminescence in minutes at room temperature is relatively rare^28^. Since the recovery rate became slower at lower temperatures, powder X-ray diffraction (PXRD) was measured at 123 K and the patterns before/after grinding were compared. The PXRD before grinding showed a distinct pattern (Supplementary Figs. 9a, 10a), which is consistent with the simulated one from the X-ray analysis of the single crystal (Supplementary Fig. 11). The pattern after grinding was the same as that before grinding and contained no new peaks (Supplementary Figs. 9b, 10b), but the intensity of the peaks became stronger after a few minutes at room temperature (Supplementary Figs. 9b–d, 10b). These observations indicate the crystal state before grinding and the amorphous state after grinding. The crystal state is recovered from the amorphous state by thermal energy at room temperature^28^. While Tani, et al. and Ito et al. reported that recrystallization of amorphous phases was promoted by remaining crystal phases for their self-recovering mechanochromic thenil^14^ and indolylbenzothiazole^29^ derivatives, respectively, it was not clear in case of *p*-**1c**. The pressed pellet prepared from the mixed powders of *p*-**1c** and KBr also showed yellow luminescence (Fig. 6c) which was maintained for a longer time than that of the ground powder. This allowed us to measure the lifetime of the new emission band, which was 168 μs (Supplementary Fig. 12) and attributed to phosphorescence.

## Conclusion

In summary, the emission wavelength of benzil in the crystal state was found to be shifted by *o*-substituents in contrast to *p*-substituents. For *o*-substituents, electron-donating MeO and electron-withdrawing F and CF_3_ groups caused blue- and red-shifts, respectively. For *m*-substituents, only the CN group showed a red-shift among the investigated substituents. The types of luminescence in the crystal states were altered by the position and type of substituents. *m*-Substituted benzils with Br, MeO, or CN and all *o*-substituted benzils exhibited fluorescence unlike pristine benzil, while *m*-substituted benzils with F or CF_3_ showed phosphorescence. The difference in the type of luminescence was explained by the El-Sayed rule and the energy gaps between S_1_ and the closest triplet state T_3_ for intersystem crossing. In addition to *o*- and *m*-substituted benzils, *p*-substituted benzil with CF_3_ was found to show fast self-recovering phosphorescence-to-phosphorescence mechanochromism at room temperature between the crystal and amorphous states. This study revealed the variety of substituent effects on the luminescence properties of benzils. Systematic screening of various properties of benzil derivatives and other organic molecules with a variety of substituents at different positions is also expected to explore potentiality of them as organic materials.

This work was supported by JSPS KAKENHI Grant Number JP20K05499 (S.H.), JST SPRING Grant Number JPMJSP2123 (M.Y.), the Fukuoka Naohiko Memorial Foundation (S.H.), and the Sumitomo Foundation (S.H.). The computations were performed using Research Center for Computational Science, Okazaki, Japan (Project: 22-IMS-C230).

## Methods

### General procedure for the synthesis of 1

In a glove box, a test tube equipped with a magnetic stir bar was charged with aryl aldehyde **3** (1.0 mmol), *tert*-butyl isonicotinate (17.9 mg, 0.10 mmol) and bis(pinacolato)diboron (B_2_pin_2_, 178 mg, 0.70 mmol). To this test tube was added cyclopentyl methyl ether (1.0 mL), and the test tube was capped with a rubber septum. The test tube was taken out from the glove box and placed in a preheated aluminum heating block. The mixture was stirred under argon atmosphere at the reflux temperature. After 6 h, the reaction mixture was cooled to room temperature. This mixture was transferred to another test tube with CH_2_Cl_2_ (3.0 mL) and stirred with 4.5 M *aq*. KHF_2_ (2.0 mL) at room temperature under air. After 3 h, the mixture was poured into water (20 mL) and extracted with CH_2_Cl_2_ three times. The combined organic layer was dried over anhydrous Na_2_SO_4_, filtered through Celite, and evaporated in vacuo. The residue was dissolved in 50% *aq*. MeOH and evaporated again. The residue was purified by SiO_2_ column chromatography to give diol **2** as a diastereomeric mixture. Diol **2** (0.10 mmol) and 2-iodoxybenzoic acid (84.0 mg, 0.30 mmol) in dimethyl sulfoxide (1.0 mL) in a test tube was stirred under air at the room temperature. After 3 h, the reaction mixture was diluted with water (1.0 mL) and extracted with diethyl ether three times. The combined organic layer was dried over anhydrous Na_2_SO_4_, filtered through Celite, and evaporated in vacuo. The residue was purified by SiO_2_ column chromatography to give substituted benzil **1**.

Other experimental procedures, characterization data of compounds, NMR spectra, reaction coordinates of calculations, and crystallographic data are included in Supplementary Methods in the Supplementary Information, Supplementary Data 1–6.

### Supplementary information

Supplementary Information

Description of Additional Supplementary Files

Supplementary Data 1

Supplementary Data 2

Supplementary Data 3

Supplementary Data 4

Supplementary Data 5

Supplementary Data 6

## Data Availability

All data are included in this article, Supplementary Information, Supplementary Data 1 (NMR spectra), Supplementary Data 2 (DFT calculations), and Supplementary Data 3–6 (crystallographic data). The X-ray crystallographic coordinates for structures reported in this Article have been deposited at the Cambridge Crystallographic Data Centre (CCDC), under deposition number CCDC-2255107 (***m*****-1c**), CCDC-2255108 (***o*****-1b**), CCDC-2255109 (***o*****-1c**), and CCDC-2255110 (***p*****-1c**). These data can be obtained free of charge from The Cambridge Crystallographic Data Centre via www.ccdc.cam.ac.uk/data_request/cif.
