# Supplementary material for: Synthesis and luminescence properties of substituted benzils
Source: Commun Chem. 2023 Nov 9;6:245. doi: 10.1038/s42004-023-01038-6 (PMC10636033; doi:10.1038/s42004-023-01038-6)
Supplement: Supplementary file 1 — Supplementary Information [file 42004_2023_1038_MOESM1_ESM.pdf]

Supplementary Information

Synthesis and luminescence properties of substituted benzils

Masamichi Yasui,[1,2] Takashi Fujihara,[3]* Hiroyoshi Ohtsu,[4] Yuki Wada,[4] Terumasa Shimada,[4] Yiying Zhu,[4] Masaki Kawano,[4] Kengo Hanaya,[1] Takeshi Sugai[1] and Shuhei Higashibayashi[1]*

[1]Faculty of Pharmacy, Keio University, 1-5-30 Shibakoen, Minato-ku, Tokyo 105-8512, Japan

[2]Department of Chemistry, Graduate School of Science, Chiba University, 1-33 Yayoi, Inage, Chiba 263-8522, Japan.

[3]Comprehensive Analysis Center for Science, Saitama University, Shimo-okubo, Sakura-ku, Saitama-city, Saitama 338-8570, Japan

[4]Department of Chemistry, School of Science, Tokyo Institute of Technology, 2-12-1 Ookayama, Meguro-ku, Tokyo 152-8550, Japan

Table of Contents

Supplementary Figures and Supplementary Tables S2

Supplementary Methods

1. General Information S12

2. Synthesis and Characterization of Compounds S13

3. X-ray Crystallographic analysis S17

Supplementary References S19

Supplementary Figures and Supplementary Tables

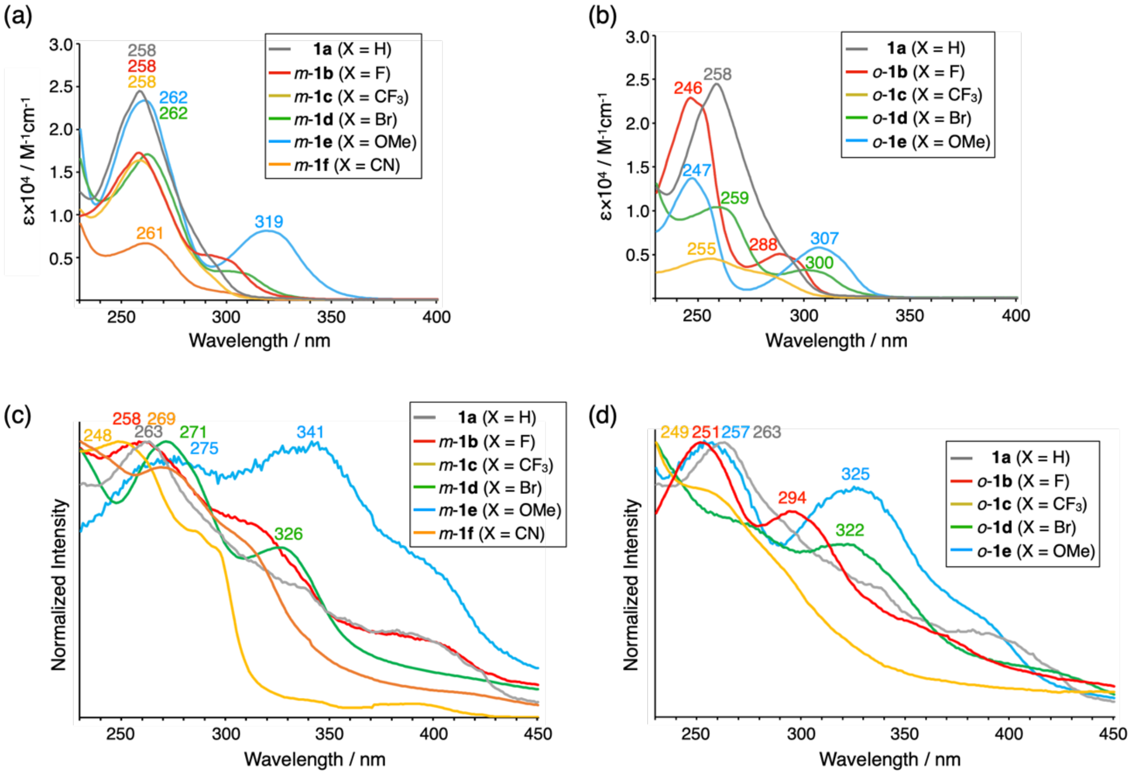

Supplementary Fig. 1 Absorption spectra of (a) *m*-**1** and (b) *o*-**1** in cyclohexane and (c) *m*-**1** and (d) *o*-**1** in crystal state.

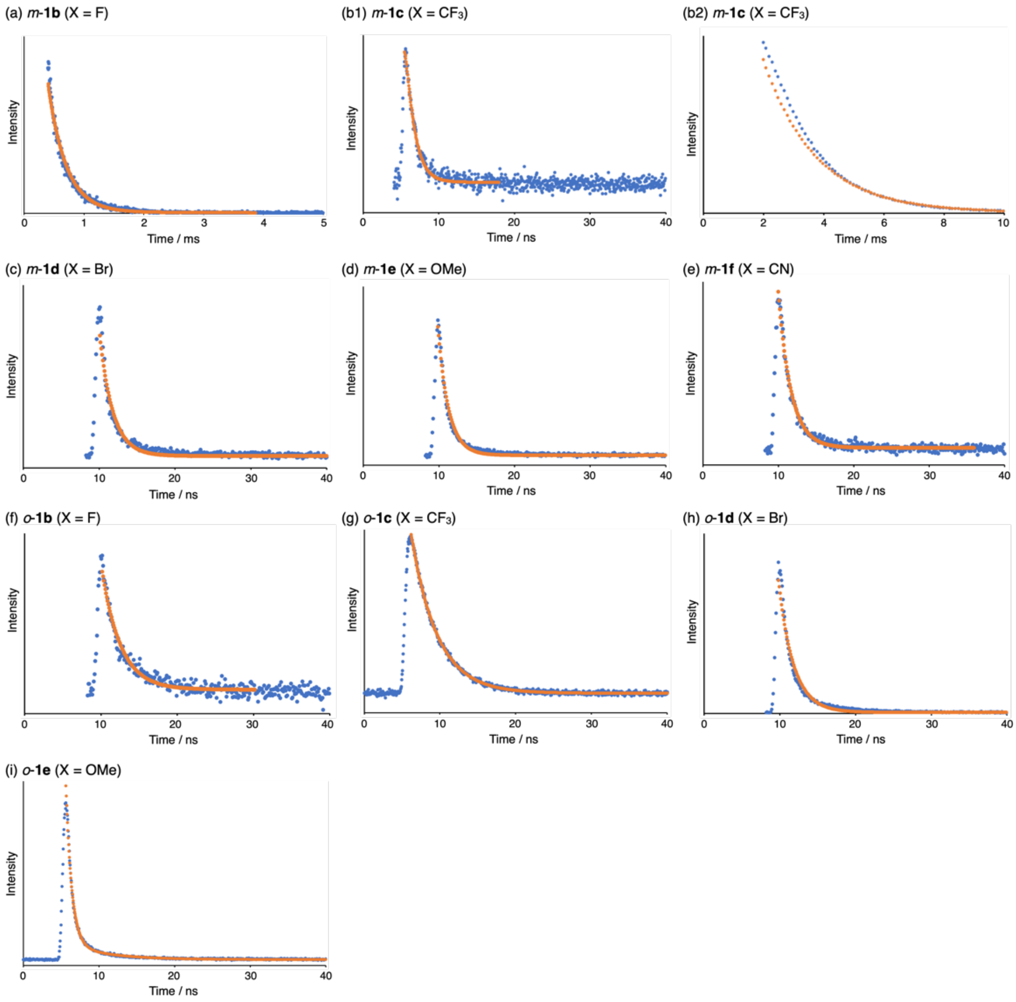

Supplementary Fig. 2 PL decay curves of *m*-**1b-f** and *o*-**1b-e** in crystal state measured by Hamamatsu Photonics Quantaurus-Tau except for (b2) by JASCO FP-6600.

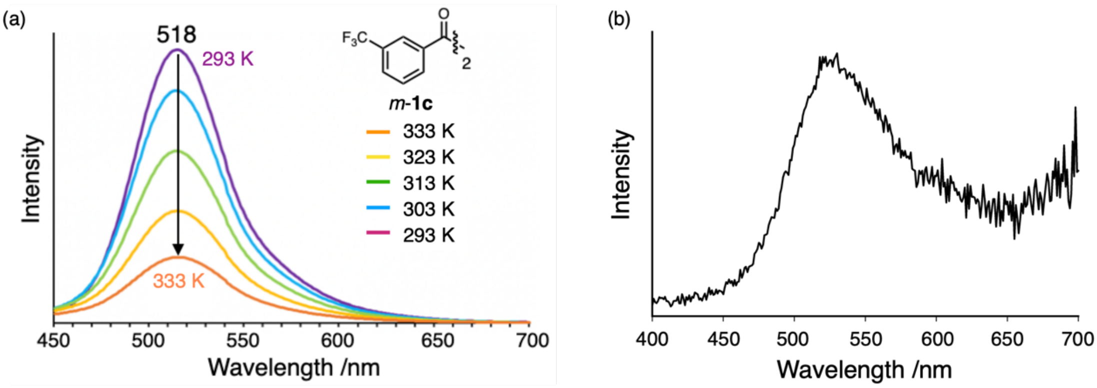

Supplementary Fig. 3 (a) Temperature-dependent emission spectra of *m*-**1c** in crystal state (excited at 365 nm). (b) Delayed (132 ms) emission spectrum of *m*-**1c** in crystal state (excited at 367 nm) at ambient temperature by JASCO FP8550.

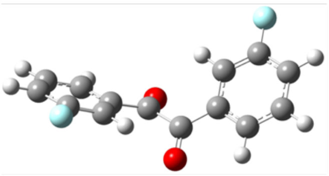

***m*-1b**
E = -888.487525 a.u.

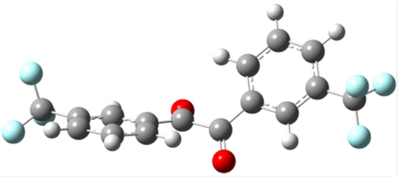

***m*-1c**
E = -1364.221716 a.u.

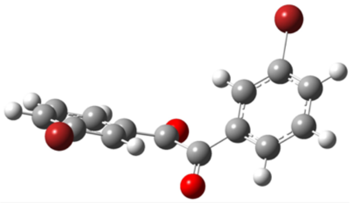

***m*-1d**
E = -5837.038677 a.u.

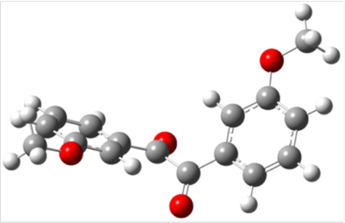

***m*-1e**
E = -918.983217 a.u.

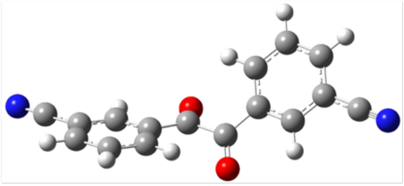

***m*-1f**
E = -874.46538 a.u.

Supplementary Fig. 4 Calculated structures and energies of *m*-**1b-f** [B3LYP/6-311+G(d,p)].

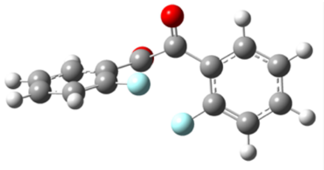

***o*-1b**
E = -888.482332 a.u.

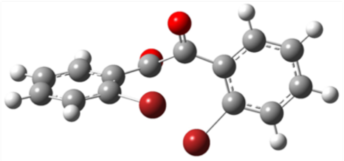

***o*-1d**
E = -5837.023194 a.u.

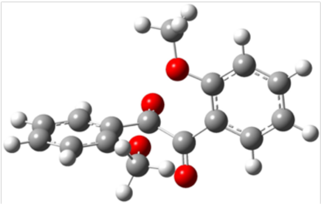

***o*-1e**
E = -918.980555 a.u.

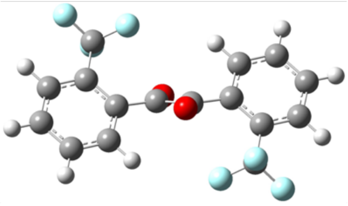

***o*-1c**
E = -1364.200434 a.u.

Supplementary Fig. 5 Calculated structures and energies of *o*-**1b-e** [B3LYP/6-311+G(d,p)].

Supplementary Table 1 Calculated energies of the excited states[a][b]

**1**	conformation	S_1	T_1	T_2	T_3
**1a**	*cis*-skew	3.21	2.68	3.09	3.13
***m*-1b** (X = F)	*cis*-skew	3.16	2.62	3.05	3.08
***m*-1c** (X = CF_3)	*cis*-skew	3.15	2.61	3.08	3.12
***m*-1d** (X = Br)	*cis*-skew	3.18	2.64	3.02	3.05
***m*-1e** (X = OMe)	*cis*-skew	3.26	2.73	3.05	3.07
***m*-1f** (X = CN)	*cis*-skew	3.12	2.58	2.98	3.02
***o*-1b** (X = F)	*cis*-skew	3.47	2.94	3.18	3.23
***o*-1c** (X = CF_3)	*trans*-planar	2.79	2.18	3.32	3.32
***o*-1d** (X = Br)	*cis*-skew	3.37	2.82	3.12	3.17
***o*-1e** (X = OMe)	*cis*-skew	3.56	3.04	3.12	3.17

[a] cam-B3LYP/6-311+G(d,p)// B3LYP/6-311+G(d,p). [b] eV.

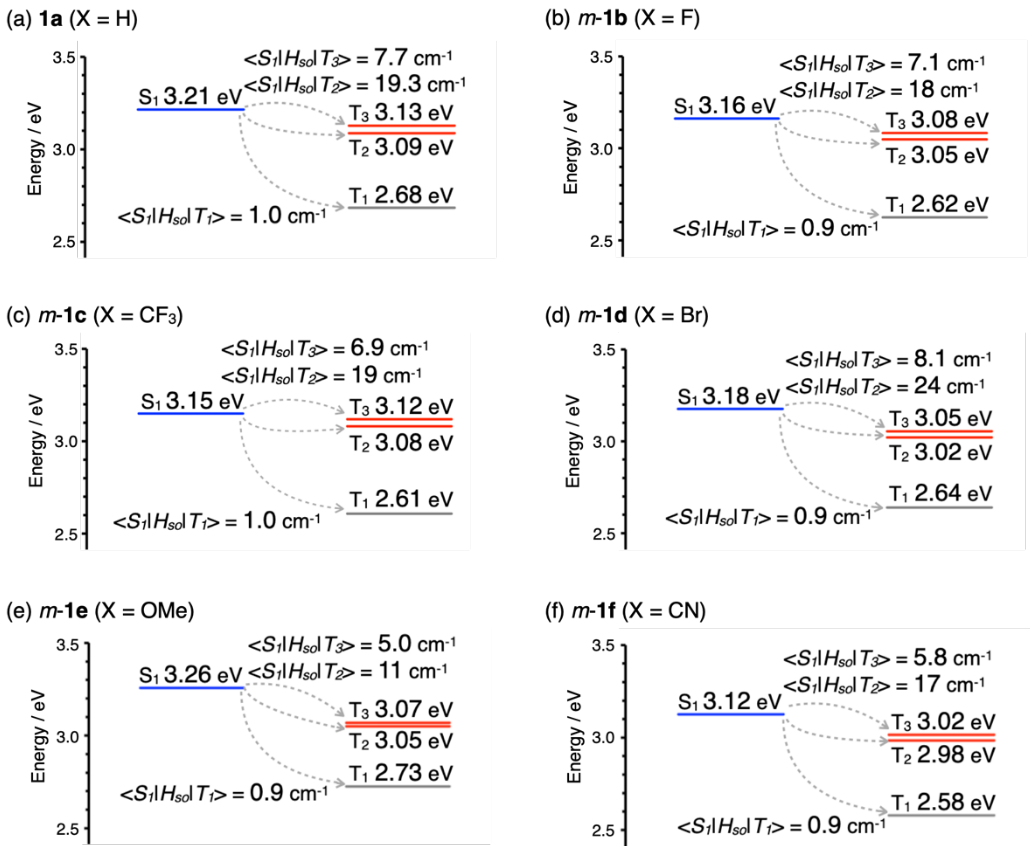

Supplementary Fig. 6 Spin-orbit TD-DFT calculation of 1a and *m*-**1b-f** [cam-B3LYP/6-311+G(d,p)/ B3LYP/6-311+G(d,p)].

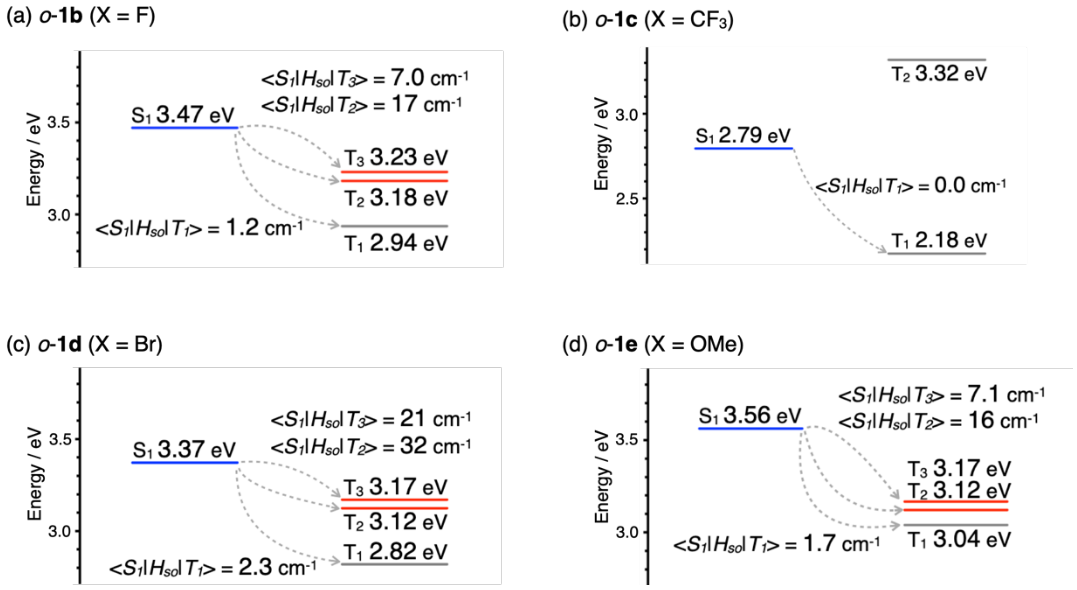

Supplementary Fig. 7 Spin-orbit TD-DFT calculation of *o*-**1b-e** [cam-B3LYP/6-311+G(d,p)/ B3LYP/6-311+G(d,p)].

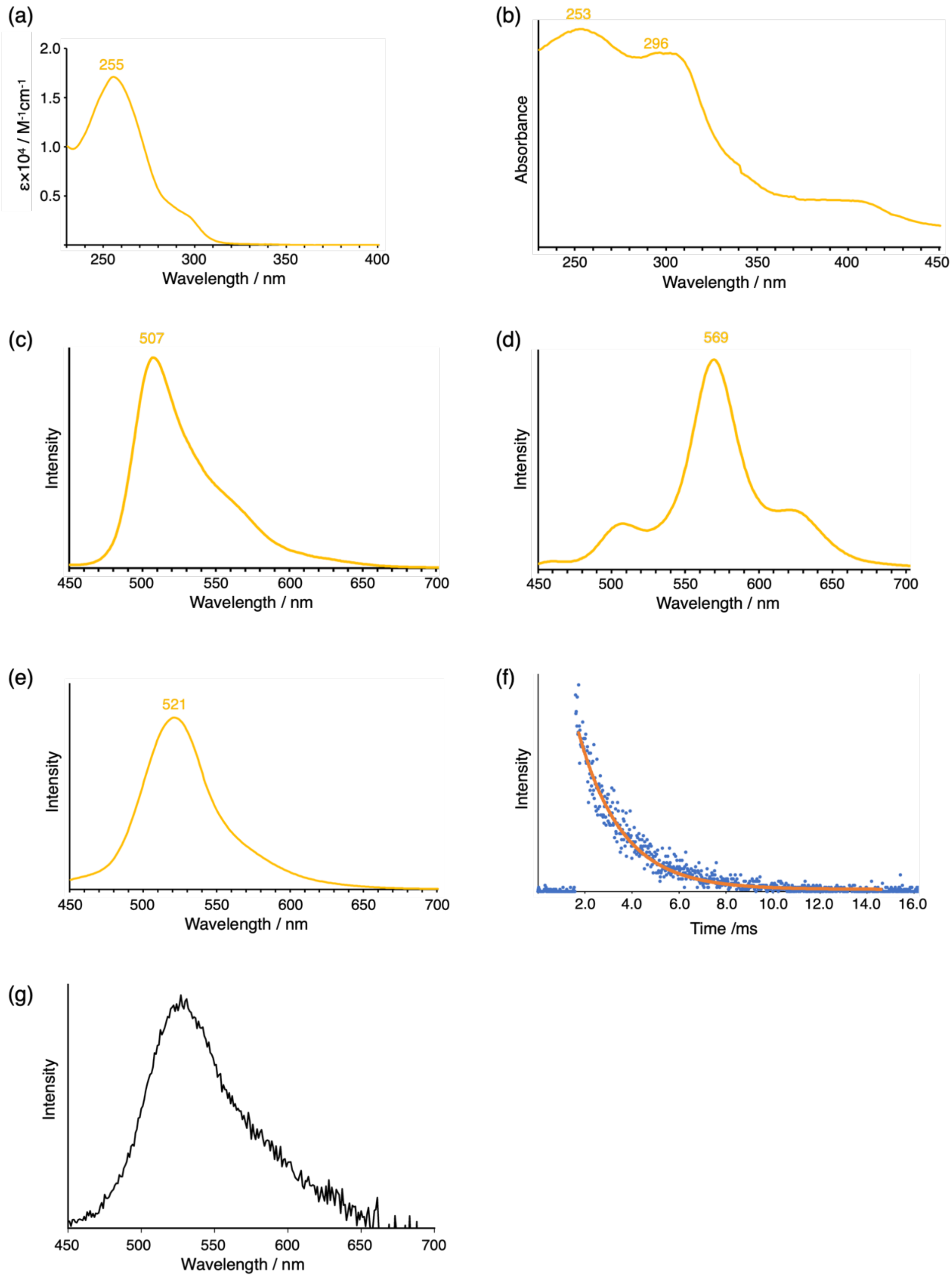

Supplementary Fig. 8 Absorption spectra of *p*-**1c** (a) in cyclohexane, (b) in crystal state. Emission spectra of *p*-**1c** (c) under air, (d) under Ar in cyclohexane (excited at 260 nm), and (e) in crystal state (excited at 260 nm). (f) PL decay curves of *p*-**1c** in crystal state at 521 nm (excited at 365 nm). (g) Delayed (132 ms) emission spectrum of *p*-**1c** in crystal state (excited at 367 nm) at ambient temperature by JASCO FP8550.

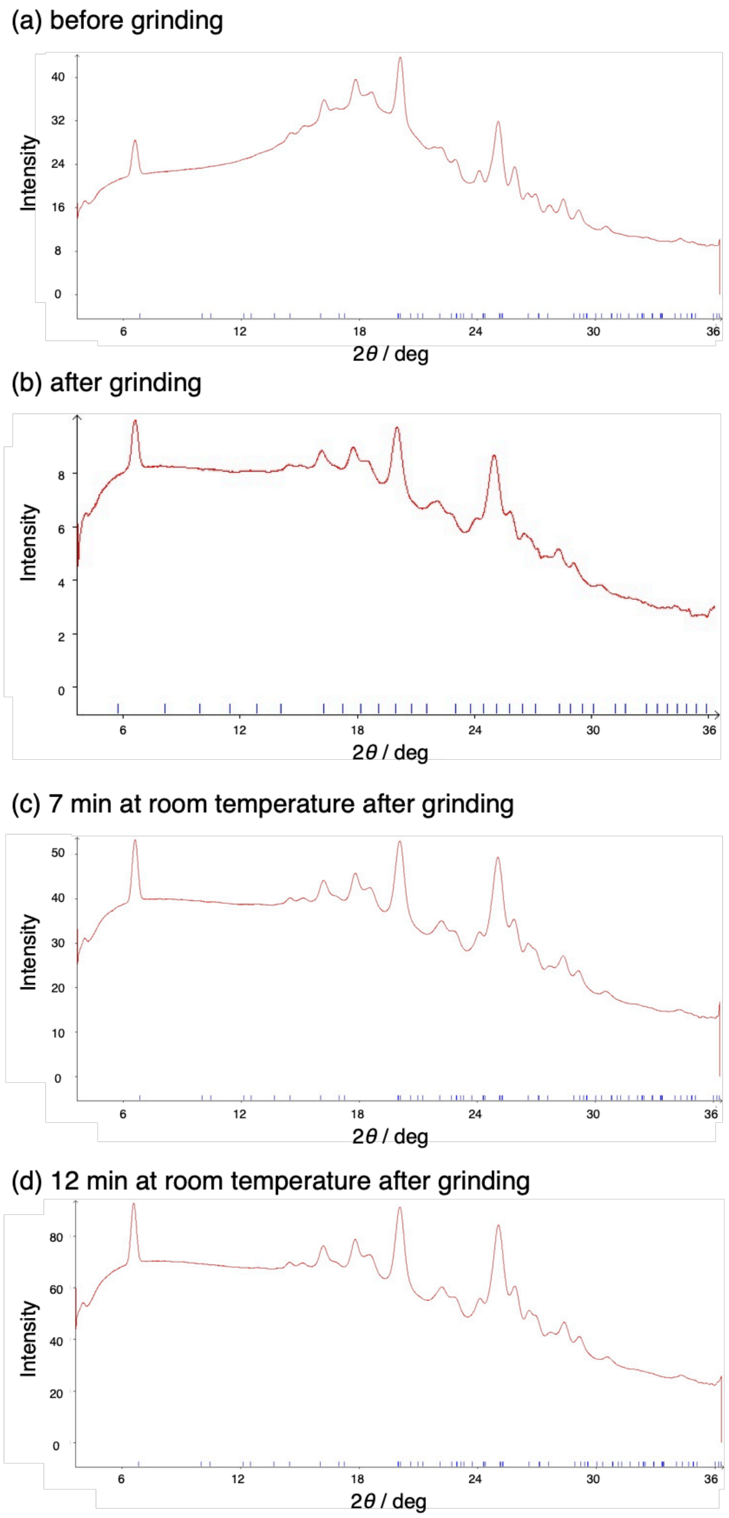

Supplementary Fig. 9 Powder X-ray diffraction patterns of *p*-**1c** measured at 123 K by a Rigaku XtaLAB Synergy-R;/DWTI APEX II; (a) before grinding, (b) after grinding, (c) 7 min at room temperature after grinding, and (d) 12 min at room temperature after grinding.

The time-dependent change of powder X-ray diffraction of the ground sample of *p*-**1c** was measured at room temperature by a Rigaku Miniflex powder diffractometer with D/teX Ultra (1D) detector (Fig. S10b). However, it took several minutes to set up the ground powders in the instrument for measurement after grinding. Since most of powders were already recovered to the crystal state, only slight increase of peaks was observed after 17 min.

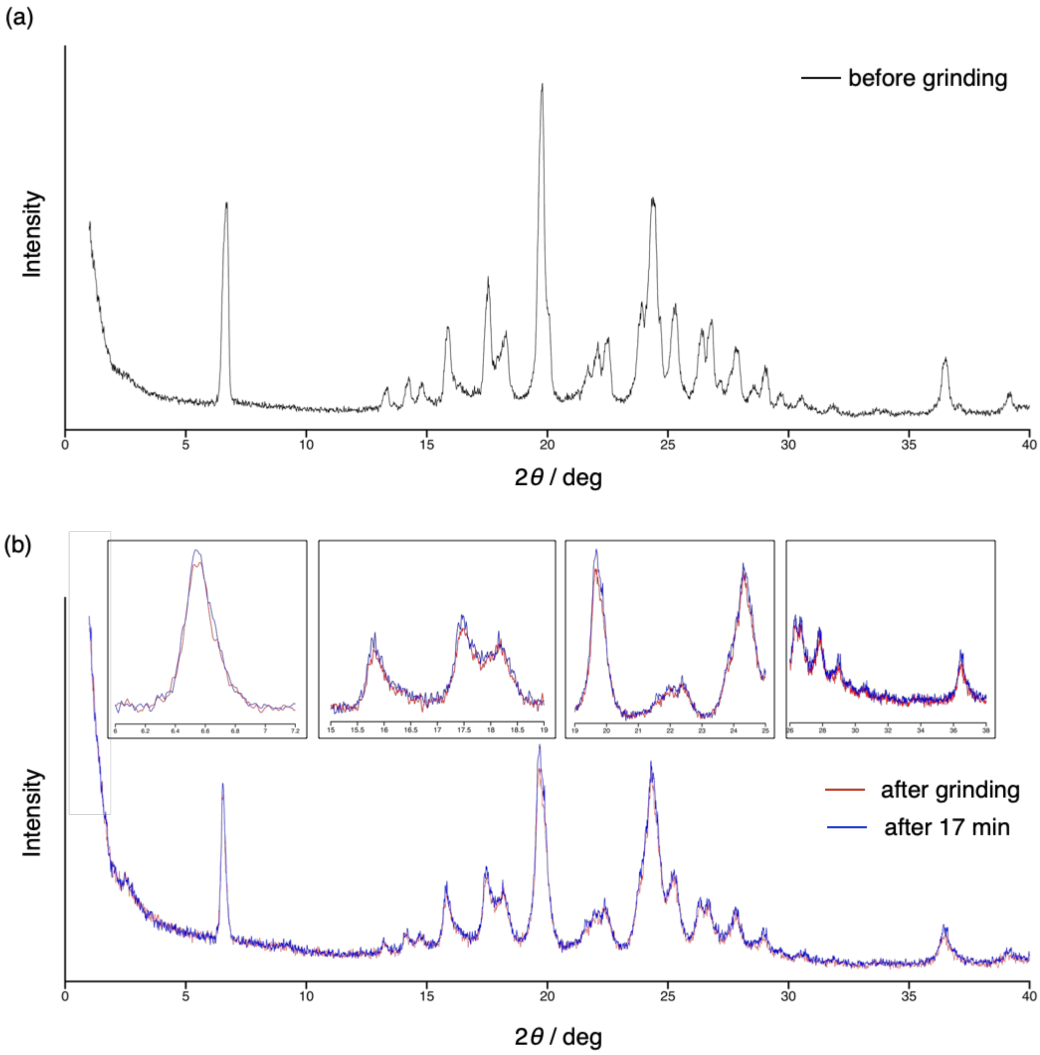

Supplementary Fig. 10 Powder X-ray diffraction patterns of *p*-**1c** measured at room temperature by a Rigaku Miniflex powder diffractometer with D/teX Ultra (1D) detector; (a) before grinding, (b) after grinding, (c) after 17 min at room temperature.

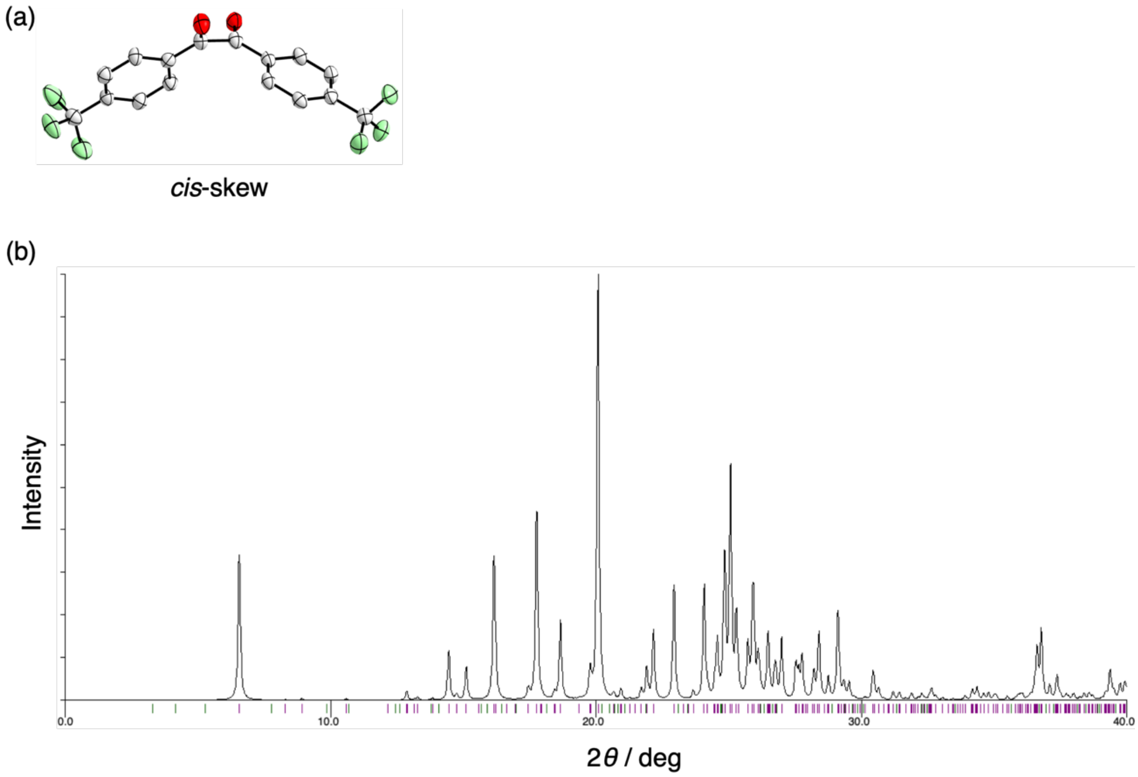

Supplementary Fig. 11 (a) ORTEP drawings of *p*-**1c** at the 50% probability level by X-ray analysis and simulated powder XRD pattern. Hydrogens are omitted for clarity.

The pressed KBr pellet of *p*-**1c** showed a new emission band around 572 nm (Fig. S12a). The measurement of the luminescence lifetime at 572 nm showed two components 168 μs (37%) and 944 μs (63%)(Fig. S12b). Judging from the intensity of the emission spectrum (Fig. 12a), the minor component 168 μs was assigned to the lifetime of the new emission. The KBr pellet was crushed and left over. After 1 week, the new emission band almost disappeared (Fig. S12c) and the luminescence lifetime at 572 nm was 1.54 ms. The lifetime of pristine emission (944 μs) in KBr pellet was shorter from the those of the powder sample of *p*-**1c** (1.86 ms) and the crushed sample (1.54 ms) of KBr pellet, which is assumed to derive from the external heavy-atom effect of KBr.[1,2]

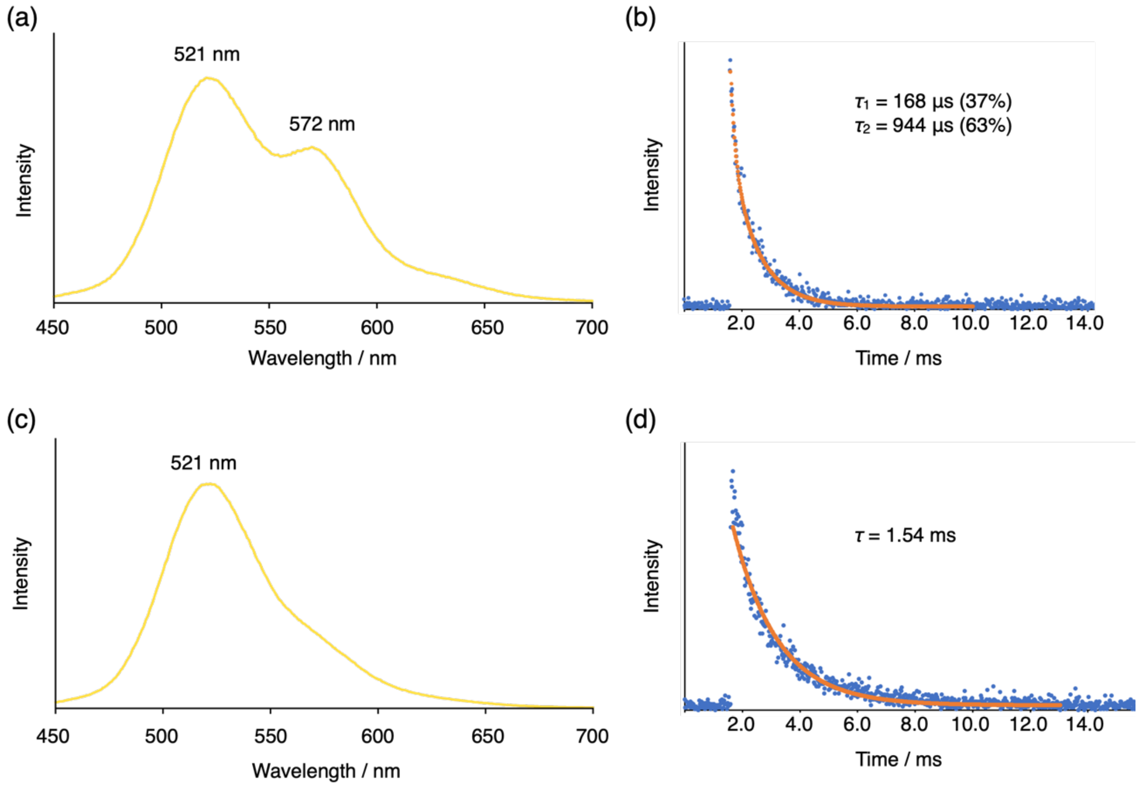

Supplementary Fig. 12 (a) Emission spectra and PL decay curve at 572 nm (excited at 365 nm) of KBr pellet of *p*-**1c**. (c) Emission spectra and PL decay curve at 572 nm (excited at 365 nm) of crushed sample of KBr pellet of *p*-**1c** after 1 week.

Supplementary Methods

1. General Information

Reagents and solvents for syntheses were commercially purchased and used as received, except 2-iodoxybenzoic acid (IBX). IBX was synthesized according to the literature procedure.[3] Air and/or moisture sensitive reactions were carried out by using dry solvents under an argon atmosphere using an argon balloon. The reactions at high temperature were performed with Organic Synthesizer, SIBATA SCIENTIFIC TECHNOLOGY Chemist Plaza CP-1000, using aluminum heating block with cooling circulator. TLC analysis was performed using Merck TLC Silica gel 60 $F254_{254}$. Flash silica gel column chromatography was performed on Wako Wakosil® C-300. Melting point was measured on an automated melting point system, MP70 Melting Point System. IR spectra were recorded on a Jasco FT/IR-4700 spectrometer with ATR PRO ONE in ATR mode using diamond prism. ^{1}H NMR spectra were measured on a Bruker spectrometer or a JEOL spectrometer at 500 MHz and ^{13}C NMR were measured on a Bruker spectrometer at 126 MHz. $CDCl_3$ was used as a solvent and the residual solvent peaks were used as an internal standard ($CDCl_3$: ^{1}H NMR: 7.26 ppm; ^{13}C NMR: 77.16 ppm). High resolution (HR) mass spectra (MS) were measured on JEOL JMS-T100LP using electrospray ionization (ESI). UV-vis absorption and PL spectra of solutions were acquired using a JASCO V-770 spectrometer and a JASCO FP-8600 spectrofluorometer, respectively. The absorption spectra of benzils (5.0×10^{-5} M) and 9,10-diphenylanthracene (DPA, 1.0×10^{-5} M) in cyclohexane solution were measured with 1 cm quartz cell at 25 °C. The fluorescence spectrum of DPA solution was measured in 1.0×10^{-7} M in order to prevent reabsorption of fluorescence. The PL spectra of the benzil (5.0×10^{-5} M) and 9,10-diphenylanthracene (DPA, 1.0×10^{-7} M) in cyclohexane solution were measured with 1 cm quartz cell at 25 °C. Photoluminescence quantum yields (PLQYs) of benzils in cyclohexane were determined by the relative method using DPA as a standard [Φ = 0.77 (under air), 1.00 (Ar atmosphere) in cyclohexane, excitation wavelength = 254 nm, 25 °C].[4] Absorption spectra of benzils in crystal state were recorded on a JASCO V-770 spectrometer in KBr discs at 25 °C. PL spectra of benzils in crystal state were recorded on a JASCO FP-8600 spectrometer with a powder sample cell block, JASCO FPA-810, at 25 °C. PLQYs of benzils in crystal state were determined by the absolute method using a JASCO FP-8600 spectrometer with an integrating sphere, JASCO ISF-834, at 25 °C. Temperature-dependent experiments were conducted using JASCO FP-8600 spectrometer with a cryostat, UNISOKU USP-203. Luminescence lifetime was measured by Hamamatsu Photonics

Quantaurus-Tau or JASCO FP-6600.

2. Synthesis and Characterization of Compounds

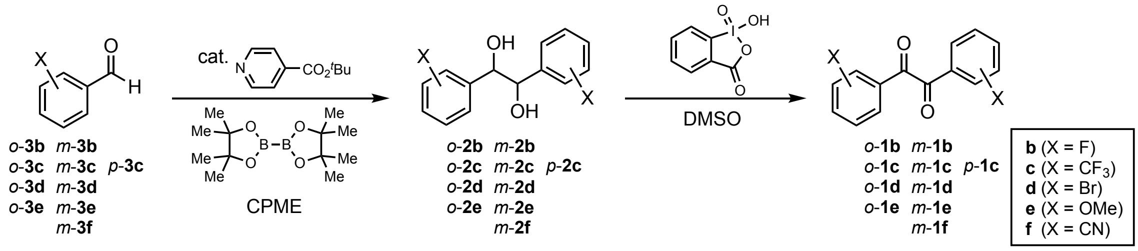

2.1 Isonicotinate-Catalyzed Pinacol Coupling

Benzil (**1a**) was commercially purchased and recrystallized from EtOH. 1,2-Bis(3-fluorophenyl)ethane-1,2-diol (***m*-2b**), 1,2-bis[3-(trifluoromethyl)phenyl]ethane-1,2-diol (***m*-2c**), 1,2-bis(3-bromophenyl)ethane-1,2-diol (***m*-2d**), 1,2-bis(3-methoxyphenyl)ethane-1,2-diol (***m*-2e**), 3,3'-(1,2-dihydroxyethane-1,2-diyl)dibenzonitrile (***m*-2f**), 1,2-bis(2-bromophenyl)ethane-1,2-diol (***o*-2d**) and 1,2-bis(2-methoxyphenyl)ethane-1,2-diol (***o*-2e**) were prepared in our previous work.[5]

1,2-Bis(2-fluorophenyl)ethane-1,2-diol (***o*-2b**), 1,2-bis[2-(trifluoromethyl)phenyl]ethane-1,2-diol (***o*-2c**) were synthesized according to the previously reported procedure. In a glove box, a test tube equipped with a magnetic stir bar was charged with aryl aldehyde **3** (1.0 mmol), *tert*-butyl isonicotinate (17.9 mg, 0.10 mmol) and bis(pinacolato)diboron (B_2pin_2, 178 mg, 0.70 mmol). To this test tube was added cyclopentyl methyl ether (CPME, 1.0 mL), and the test tube was capped with a rubber septum. The test tube was taken out from the glove box and placed in a preheated aluminum heating block. The mixture was stirred under argon atmosphere at the reflux temperature. After 6 h, the reaction mixture was cooled to room temperature. This mixture was transferred to another test tube with CH_2Cl_2 (3.0 mL) and stirred with 4.5 M *aq.* KHF_2 (2.0 mL) at room temperature under air. After 3 h, the mixture was poured into water (20 mL) and extracted with CH_2Cl_2 (20 mL × 3). The combined organic layer was dried over anhydrous Na_2SO_4 and filtered through Celite, and the filtrate was evaporated *in vacuo*. The residue was dissolved in 50% *aq.* MeOH and evaporated again. The residue was purified by SiO_2 column chromatography to give diol **2** as a diastereomeric mixture.

1,2-Bis(2-fluorophenyl)ethane-1,2-diol (*o*-2b)

The reaction was carried out with 2-fluorobenzaldehyde (124 mg, 1.0 mmol). Purification by SiO_2 column chromatography (0-25% EtOAc/hexane) gave ***o*-2b** (46.5 mg, 38% yield, *dr*=1:1). The ^{1}H NMR spectrum was identical with that in the literature.[6]

^{1}H NMR ($CDCl_3$): 7.44 (ddd, J = 7.4, 7.4, 1.7 Hz, 2H), 7.26-7.18 (m, 6H), 7.10 (ddd, J = 7.5, 7.5, 1.1 Hz, 2H), 7.03 (ddd, J = 7.5, 7.5, 1.1 Hz, 2H), 6.93-6.88 (m, 4H), 5.36 (s, 2H), 5.14 (s, 2H), 2.87 (s, 2H), 2.64 (s, 2H) ppm.

1,2-Bis{2-(trifluoromethyl)phenyl}ethane-1,2-diol (*o*-2c)

The reaction was carried out with 2-(trifluoromethyl)benzaldehyde (174 mg, 1.0 mmol). Purification by SiO_2 column chromatography (0-25% EtOAc/hexane) gave ***o*-2c** (132 mg, 74% yield, *dr*=1:1). The ^{1}H NMR spectrum was identical with that in the literature.[7,8]

^{1}H NMR ($CDCl_3$): 7.90 (d, J = 8.2 Hz, 2H), 7.61-7.56 (m, 10H), 7.50 (dd, J = 8.2, 8.2 Hz, 2H), 7.38 (dd, J = 8.2, 8.2 Hz, 2H), 5.43 (s, 2H), 5.37 (s, 2H), 3.05 (s, 2H), 2.53 (s, 2H) ppm.

1,2-Bis{4-(trifluoromethyl)phenyl}ethane-1,2-diol (*p*-2c)

The reaction was carried out with 4-(trifluoromethyl)benzaldehyde (174 mg, 1.0 mmol). Purification by SiO_2 column chromatography (0-25% EtOAc/hexane) gave ***p*-2c** (118 mg, 68% yield, *dr*=1:1). The ^{1}H NMR spectrum was identical with that in the literature.[9]

^{1}H NMR ($CDCl_3$): 7.55 (d, J = 8.0 Hz, 4H), 7.52 (d, J = 8.1 Hz, 4H), 7.30 (d, J = 8.0 Hz, 4H), 7.24 (d, J = 8.1 Hz, 4H), 4.97 (s, 2H), 4.76 (s, 2H), 2.95 (s, 2H), 2.43 (s, 2H) ppm.

2.2 Synthesis of benzils 1

The mixture of diols **2** (0.10 mmol) and IBX (84.0 mg, 0.30 mmol) in dimethyl sulfoxide (1.0 mL) in a test tube was stirred under air at the room temperature. After 3 h, the reaction mixture was diluted with water (1.0 mL) and extracted with diethyl ether (3.0 mL x 3). The combined organic layer was dried over anhydrous Na_2SO_4 and filtered through Celite, and the filtrate was evaporated *in vacuo*. The residue was purified by SiO_2 column chromatography to give substituted benzils **1**.

1,2-Bis(3-fluorophenyl)ethane-1,2-dione (*m*-1b)

The reaction was carried out with diol ***m*-2b** (25.0 mg, 0.10 mmol). Purification by SiO_2 column chromatography (0-30% EtOAc/hexane) gave ***m*-1b** (18.9 mg, 77% yield) as a colorless solid. The

^{1}H NMR spectrum was identical with that in the literature.[10] ^{1}H NMR ($CDCl_3$): 7.74-7.72 (m, 2H), 7.71-7.69 (m, 2H), 7.51 (ddd, J = 8.1, 8.1, 5.4 Hz, 2H), 7.38 (ddd, J = 8.1, 8.1, 2.6, 1.0 Hz, 2H) ppm.

1,2-Bis{3-(trifluoromethyl)phenyl}ethane-1,2-dione (*m*-1c)

The reaction was carried out with diol ***m*-2c** (35.0 mg, 0.10 mmol). Purification by SiO_2 column chromatography (0-30% EtOAc/hexane) gave ***m*-1c** (23.7 mg, 68% yield) as a pale yellow solid. The ^{1}H NMR spectrum was identical with that in the literature.[9] ^{1}H NMR ($CDCl_3$): 8.30 (s, 2H), 8.18 (d, J = 7.8 Hz, 2H), 7.95 (d, J = 7.8 Hz, 2H), 7.70 (dd, J = 7.8 Hz, 2H) ppm.

1,2-Bis(3-bromophenyl)ethane-1,2-dione (*m*-1d)

The reaction was carried out with diol ***m*-2d** (37.2 mg, 0.10 mmol). Purification by SiO_2 column chromatography (0-20% EtOAc/hexane) gave ***m*-1d** (27.1 mg, 73% yield) as a pale yellow solid. The ^{1}H NMR spectrum was identical with that in the literature.[11] ^{1}H NMR ($CDCl_3$): 8.12 (dd, J = 1.7, 1.7 Hz, 2H), 7.89-7.87 (m, 2H), 7.81-7.79 (m, 2H), 7.41 (dd, J = 8.0, 8.0, 2H) ppm.

1,2-Bis(3-methoxyphenyl)ethane-1,2-dione (*m*-1e)

The reaction was carried out with diol ***m*-2e** (27.4 mg, 0.10 mmol). Purification by SiO_2 column chromatography (0-20% EtOAc/hexane) gave ***m*-1e** (24.9 mg, 92% yield) as a pale yellow solid. The ^{1}H NMR spectrum was identical with that in the literature.[12] ^{1}H NMR ($CDCl_3$): 7.54-7.53 (m, 2H), 7.47 (ddd, J = 7.6, 1.2, 1.2 Hz, 2H), 7.40 (dd, J = 7.6, 7.6 Hz, 2H), 7.20 (ddd, J = 7.6, 2.5, 1.2 Hz, 2H), 3.87 (s, 6H) ppm.

3,3'-Oxalyldibenzonitrile (*m*-1f)

The reaction was carried out with diol ***m*-2f** (26.0 mg, 0.10 mmol). Purification by SiO_2 column chromatography (0-20% EtOAc/hexane) gave ***m*-1f** (19.6 mg, 77% yield) as a pale yellow solid.
Mp: 178.6-179.4 ºC. IR (ATR): ν 3079, 2359, 2341, 2234, 1670, 1598, 1570, 1478, 1430, 1294, 1220, 1144, 998, 957, 926, 897, 814, 773, 720, 673, 646 cm^{-1}. ^{1}H NMR ($CDCl_3$): 8.31-8.30 (m, 2H), 8.25 (ddd, J = 7.9, 1.4, 1.4 Hz, 2H), 7.97 (ddd, J = 7.9, 1.4, 1.4 Hz, 2H), 7.71 (ddd, J = 7.9, 7.9, 0.5 Hz, 2H). ^{13}C NMR ($CDCl_3$): 190.0, 138.0, 133.9, 133.8, 133.4, 130.4, 117.4, 114.1 ppm. HRMS (*m*/*z*) for $C_{16}H_8N_2NaO_2$ (MNa^+): Calculated 283.0484, found 283.0490.

1,2-Bis(2-fluorophenyl)ethane-1,2-dione (*o*-1b)

The reaction was carried out with diol ***o*-1b** (25.0 mg, 0.10 mmol). Purification by SiO_2 column chromatography (0-30% EtOAc/hexane) gave ***o*-1b** (19.1 mg, 77% yield) as a colorless solid. The ^1H NMR spectrum was identical with that in the literature.[10] ^1H NMR ($CDCl_3$): 8.09-8.06 (m, 2H), 7.68-7.63 (m, 2H), 7.35 (ddd, J = 7.8, 7.8, 1.0, 2H), 7.17-7.13 (m, 2H) ppm.

1,2-Bis{2-(trifluoromethyl)phenyl}ethane-1,2-dione (*o*-1c)

The reaction was carried out with diol ***o*-2c** (35.0 mg, 0.10 mmol). Purification by SiO_2 column chromatography (10-30% EtOAc/hexane) gave ***o*-1c** (17.7 mg, 52% yield) as a yellow solid.
Mp: 92.2-93.0 ºC. IR (ATR): ν 2922, 2853, 2341, 1709, 1580, 1447, 1315, 1271, 1220, 1166, 1122, 1107, 1061, 1032, 965, 888, 851, 746, 637 cm^{-1}. ^1H NMR ($CDCl_3$): 7.84-7.81 (m, 2H), 7.74-7.69 (m, 4H), 7.68-7.65 (m, 2H) ppm. ^{13}C NMR ($CDCl_3$): 190.3, 133.7, 132.1, 131.9, 130.1, 129.2 (J = 32.8 Hz), 127.2 (J = 5.2 Hz), 123.6 (J = 274.1 Hz) ppm. HRMS (*m*/*z*) for $C_{16}H_8F_6NaO_2$ (MNa^+): Calculated 369.0326, found 369.0306.

1,2-Bis(2-bromophenyl)ethane-1,2-dione (*o*-1d)

The reaction was carried out with diol ***o*-2d** (37.2 mg, 0.10 mmol). Purification by SiO_2 column chromatography (0-20% EtOAc/hexane) gave ***o*-1d** (21.3 mg, 57% yield) as a pale yellow solid.
Mp: 108.5-109.5 ºC. IR (ATR): ν 3065, 2922, 2359, 2340, 1668, 1566, 1464, 1418, 1301, 1271, 1220, 1188, 1067, 992, 909, 772, 670, 641 cm^{-1}. ^1H NMR ($CDCl_3$): 7.99-7.97 (m, 2H), 7.69-7.67 (m, 2H), 7.50- 7.43 (m, 4H) ppm. ^{13}C NMR ($CDCl_3$): 191.3, 134.7, 134.6, 134.3, 133.5, 127.8, 123.4 ppm. HRMS (*m*/*z*) for $C_{14}H_8{}^{79}Br_2NaO_2$ (MNa^+): Calculated 388.8789, found 388.8800.

1,2-Bis(2-methoxyphenyl)ethane-1,2-dione (*o*-1e)

The reaction was carried out with diol ***o*-2e** (27.4 mg, 0.10 mmol). Purification by SiO_2 column chromatography (0-50% EtOAc/hexane) gave ***o*-1e** (23.8 mg, 88% yield) as a pale yellow solid. The ^1H NMR spectrum was identical with that in the literature.[12] ^1H NMR ($CDCl_3$): 8.09 (dd, J = 7.8, 1.8 Hz, 2H), 7.58-7.55 (m, 2H), 7.12 (ddd, J = 7.8, 7.8, 1.0 Hz, 2H), 6.95 (d, J = 7.8 Hz, 2H), 3.59 (s, 6H) ppm.

1,2-Bis{4-(trifluoromethyl)phenyl}ethane-1,2-dione (*p*-1c)

The reaction was carried out with diol ***p*-2c** (35.0 mg, 0.10 mmol). Purification by SiO_2 column chromatography (0-25% EtOAc/hexane) gave ***o*-1c** (30.0 mg, 87% yield) as a yellowish-green solid. The ^{1}H NMR spectrum was identical with that in the literature.[10,13]

^{1}H NMR ($CDCl_3$): 8.12 (d, J = 8.1 Hz, 4H), 7.81 (d, J = 8.1 Hz, 4H) ppm.

3. X-ray Crystallographic Analysis

X-ray crystallographic analyses of single crystals of *m*-**1c**, *o*-**1b**, and *o*-**1c** were performed on a Rigaku Synergy-R/DWTI APEX II instrument with a Hypix-6000HE detector (Cu-$K\alpha$, λ=1.514184 Å, T=123.15 K). The structures were solved by Dual space methods (SHELXT-2018) and refined by full-matrix least squares calculations on F^2 (SHELXL-2018) using the SHELX-TL program package. All non-hydrogen atoms were refined with anisotropic displacement parameters. All hydrogen atoms were created with ideal geometry and refined using a riding model. Crystallographic data have been deposited with Cambridge Crystallographic Data Centre: Deposition number CCDC-2255107 (***m*-1c**), CCDC-2255108 (***o*-1b**), CCDC-2255109 (***o*-1c**), and CCDC-2255110 (***p*-1c**). Copies of the data can be obtained free of charge via http://www.ccdc.cam.ac.uk/conts/retrieving.html (or from the Cambridge Crystallographic Data Centre, 12, Union Road, Cambridge, CB2 1EZ, UK; Fax: +44 1223 336033; e-mail: deposit@ccdc.cam.ac.uk).

The single crystal of *m*-**1c** was obtained by the slow vaporization from DMSO/H_2O solution. Crystal data of *m*-1**c**: $C_{16}H_8F_6O_2$, colorless, 0.09 × 0.04×0.03 mm^3, monoclinic, space group P12$_1$/c1 (No. 14), a = 13.957(1), b = 8.2530(5), c = 12.0509(6) Å, α = 90°, β = 99.433(6)°, γ = 90°, V = 1369.34(15) Å^3, ρ_{calcd} = 1.679 g/cm^3, Z = 4, 2413 reflections measured, R_1 = 0.0786 [$I > 2\sigma(I)$], and wR_2 = 0.2304 (all data), GOF = 1.017.

The single crystal of *o*-**1b** was obtained by the slow vaporization from EtOH/H_2O solution. Crystal data of *o*-**1b**: $C_{14}H_8F_2O_2$, colorless, 0.17×0.05×0.04 mm^3, triclinic (No. 2), space group P-1, a = 7.0042(3), b = 7.4619(4), c = 11.5458(4) Å, α = 78.404(4)°, β = 82.273(3)°, γ = 70.880(4)°, V = 556.98(4) Å^3, ρ_{calcd} = 1.468 g/cm^3, Z = 2, 2142 reflections measured, R_1 = 0.0408 [$I > 2\sigma(I)$], and wR_2 = 0.1173 (all data), GOF = 1.076.

The single crystal of *o*-**1c** was obtained by the slow vaporization from DMSO/H_2O solution. Crystal data of *o*-**1c**: $C_{16}H_8F_6O_2$, yellow, 0.14×0.11×0.08 mm^3, monoclinic (No. 14), space group P12$_1$/c1, a = 7.82524(17), b = 13.6824(3), c = 7.24532(14) Å, α = 90°, β = 116.520(3)°, γ = 90°, V = 694.12(3) Å^3, ρ_{calcd} = 1.657 g/cm^3, Z = 2, 1452 reflections measured, R_1 = 0.0360 [$I > 2\sigma(I)$], and wR_2 = 0.0991 (all data), GOF = 1.064.

The single crystal of *p*-**1c** was obtained by the slow vaporization from EtOH/H_2O solution. Crystal data of *p*-**1c**: $C_{48}H_{24}F_{18}O_6$, yellow, 0.022×0.017×0.006 mm^3, orthorhombic (No. 29), space group $Pca2_1$, a = 21.2933(4), b = 7.2637(2), c = 26.9079(5) Å, α = 90°, β = 90, γ = 90°, V = 4161.79(16) $Å^3$, ρ_{calcd} = 1.658 g/cm^3, Z = 4, 7478 reflections measured, R_1 = 0.0565 [$I > 2\sigma(I)$], and wR_2 = 0.1527 (all data), GOF = 1.073.

Powder X-ray diffraction patterns were measured on a Rigaku Synergy-R/DWTI APEX II instrument with a Hypix-6000HE detector (Cu-$K\alpha$, λ=1.514184 Å, T=123.15 K) or a Rigaku Miniflex powder diffractometer with D/teX Ultra (1D) detector (Cu-$K\alpha$, λ=1.514184 Å, room temperature).

Supplementary References

1. McGlynn, S. P., Reynolds, M. J., Daigre, G. W. & Christodoyleas, N. D. The external heavy-atom spin-orbital coupling effect. III. Phosphorescence spectra and lifetimes of externally perturbed naphthalenes. *J. Phys. Chem.* **66**, 2499–2505 (1962).
2. Yan, Z., Lin, X., Sun, S., Ma, X. & Tian, H. Activating room-temperature phosphorescence of organic luminophores via external heavy-atom effect and rigidity of ionic polymer matrix. *Angew. Chem. Int. Ed.* **60**, 19735–19739 (2021).
3. Frigerio, M., Santagostino, M. & Sputore, S. A user-friendly entry to 2-iodoxybenzoic acid (IBX). *J. Org. Chem.* **64**, 4537–4538 (1999).
4. Heinrich, G., Schoof, S. & Gusten, H. 9,10-Diphenylanthracene as a fluorescence quantum yield standard. *J. Photochem.* **3**, 315-320 (1974/75)
5. Yasui, M., Hanaya, K., Sugai, T. & Higashibayashi, S. Metal-free thermal organocatalytic pinacol coupling of arylaldehydes using an isonicotinate catalyst with bis(pinacolato)diboron. *RSC Adv.* **11**, 24652–24655 (2021).
6. Wang, C., Pan, Y. & Wu, A. $InCl_3$/Al mediated pinacol coupling reactions of aldehydes and ketones in aqueous media. *Tetrahedron* **63**, 429–434 (2007).
7. Broeker, J., Knollmueller, M. & Gaertner, P. Chiral linker. Part 3: Synthesis and evaluation of aryl substituted *m*-hydrobenzoins as solid supported open chain chiral auxiliaries for the diastereoselective reduction of α-keto esters. *Tetrahedron: Asymmetry* **17**, 2413–2429 (2006).
8. Rauniyar, V., Zhai, H. & Hall, D. G. Catalytic enantioselective allyl- and crotylboration of aldehydes using chiral diol•$SnCl_4$ complexes. Optimization, substrate scope and mechanistic investigations. *J. Am. Chem. Soc.* **130**, 8481–8490 (2008).
9. Ohe, T., Ohse, T., Mori, K., Ohtaka, S. & Uemura, S. Indium-catalyzed cross-coupling reactions between α,β-unsaturated carbonyl compounds and aromatic aldehydes. *Bull. Chem. Soc. Jpn.* **76**, 1823–1827 (2003).
10. Hicks, L. D. *et al.* Analysis of the inhibition of mammalian carboxylesterases by novel fluorobenzoins and fluorobenzils. *Bioorg. Med. Chem.* **15**, 3801–3817 (2007).
11. Li, J.-T. & Sun, X.-L. An efficient synthesis of benzils from hydrobenzoins by CrO_3-NH_4Cl under ultrasound irradiation. *Lett. Org. Chem.* **3**, 842–844 (2006).
12. Alamsetti, S. K., Mannam, S., Mutupandi, P. & Sekar, G. Galactose oxidase model: Biomimetic enantiomer-differentiating oxidation of alcohols by a chiral copper complex. *Chem. – Eur. J.* **15**, 1086–1090 (2009).
13. Braña, M. F. *et al.* Pyrazolo[3,4- *c*]pyridazines as novel and selective inhibitors of cyclin-dependent kinases. *J. Med. Chem.* **48**, 6843–6854 (2005).
