# Supplementary material for: Synthesis and luminescence properties of substituted benzils
Source: Commun Chem. 2023 Nov 9;6:245. doi: 10.1038/s42004-023-01038-6 (PMC10636033; doi:10.1038/s42004-023-01038-6)
Supplement: Supplementary file 2 — Description of Additional Supplementary Files [file 42004_2023_1038_MOESM2_ESM.pdf]

Description of Additional Supplementary Files

File name: Supplementary Data 1

Description: NMR spectra

File name: Supplementary Data 2

Description: Reaction coordinates of DFT calculations

File name: Supplementary Data 3

Description: Crystallographic data of ***m*-1c**

File name: Supplementary Data 4

Description: Crystallographic data of ***o*-1b**

File name: Supplementary Data 5

Description: Crystallographic data of ***o*-1c**

File name: Supplementary Data 6

Description: Crystallographic data of ***p*-1c**
