# Supplementary material for: Synthesis and luminescence properties of substituted benzils
Source: Commun Chem. 2023 Nov 9;6:245. doi: 10.1038/s42004-023-01038-6 (PMC10636033; doi:10.1038/s42004-023-01038-6)

Supplementary Data 1

Synthesis and luminescence properties of substituted benzils

Masamichi Yasui,[1,2] Takashi Fujihara,[3]* Hiroyoshi Ohtsu,[4] Yuki Wada,[4] Terumasa Shimada,[4] Yiying Zhu,[4] Masaki Kawano,[4] Kengo Hanaya,[1] Takeshi Sugai[1] and Shuhei Higashibayashi[1]*

[1]Faculty of Pharmacy, Keio University, 1-5-30 Shibakoen, Minato-ku, Tokyo 105-8512, Japan

[2]Department of Chemistry, Graduate School of Science, Chiba University, 1-33 Yayoi, Inage, Chiba 263-8522, Japan.

[3]Comprehensive Analysis Center for Science, Saitama University, Shimo-okubo, Sakura-ku, Saitama-city, Saitama 338-8570, Japan

[4]Department of Chemistry, School of Science, Tokyo Institute of Technology, 2-12-1 Ookayama, Meguro-ku, Tokyo 152-8550, Japan

NMR Spectra

^{1}H NMR (500 MHz, $CDCl_3$) spectrum of compound *o*-**2b**

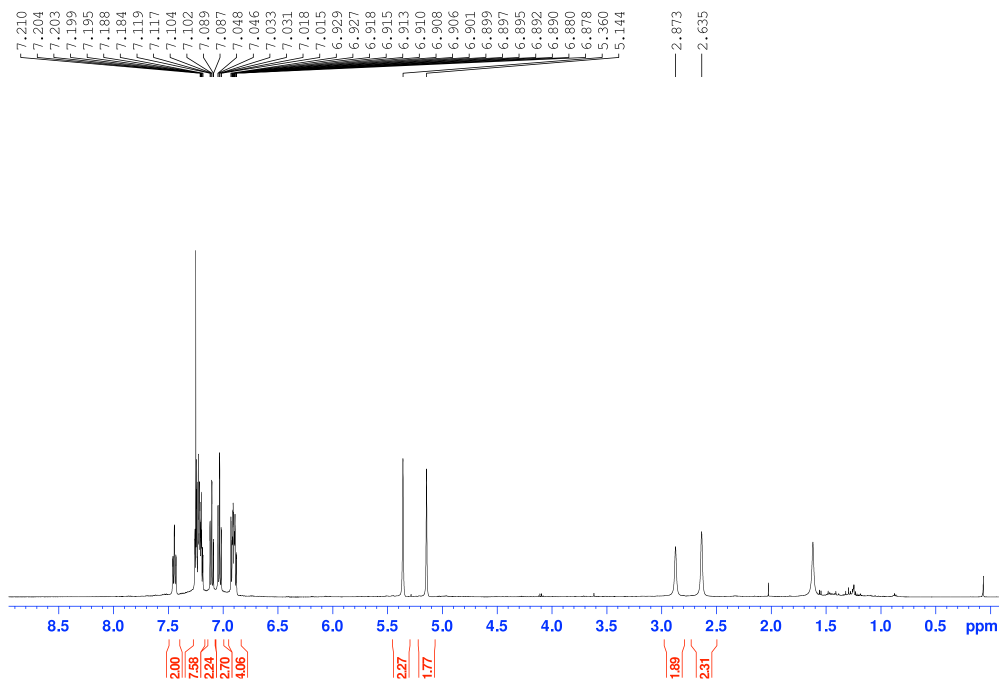

^{1}H NMR (500 MHz, $CDCl_3$) spectrum of compound *o*-**2c**

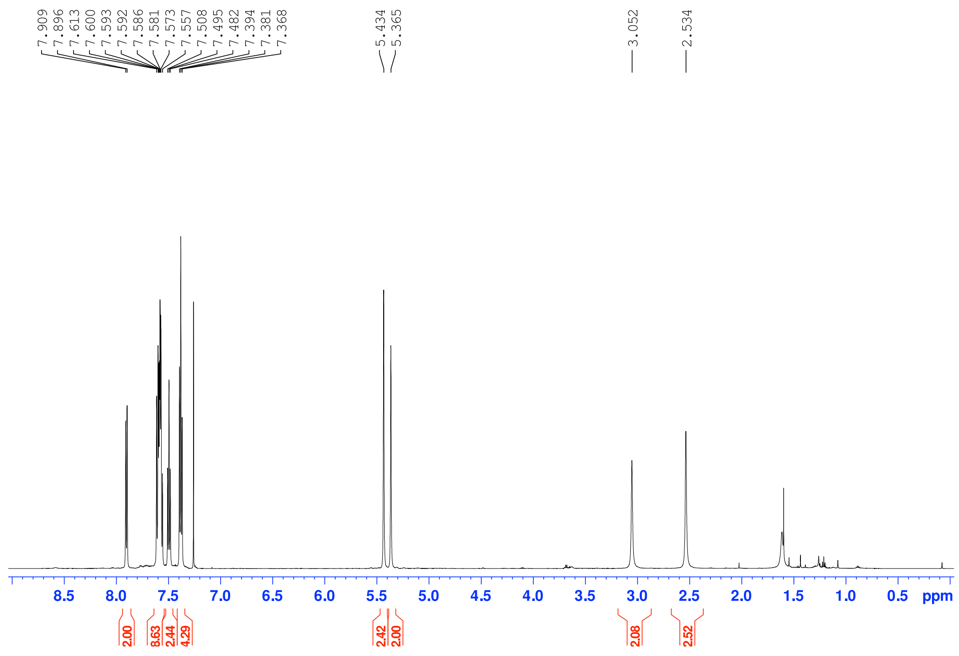

^{1}H NMR (500 MHz, $CDCl_3$) spectrum of compound *p*-**2c**

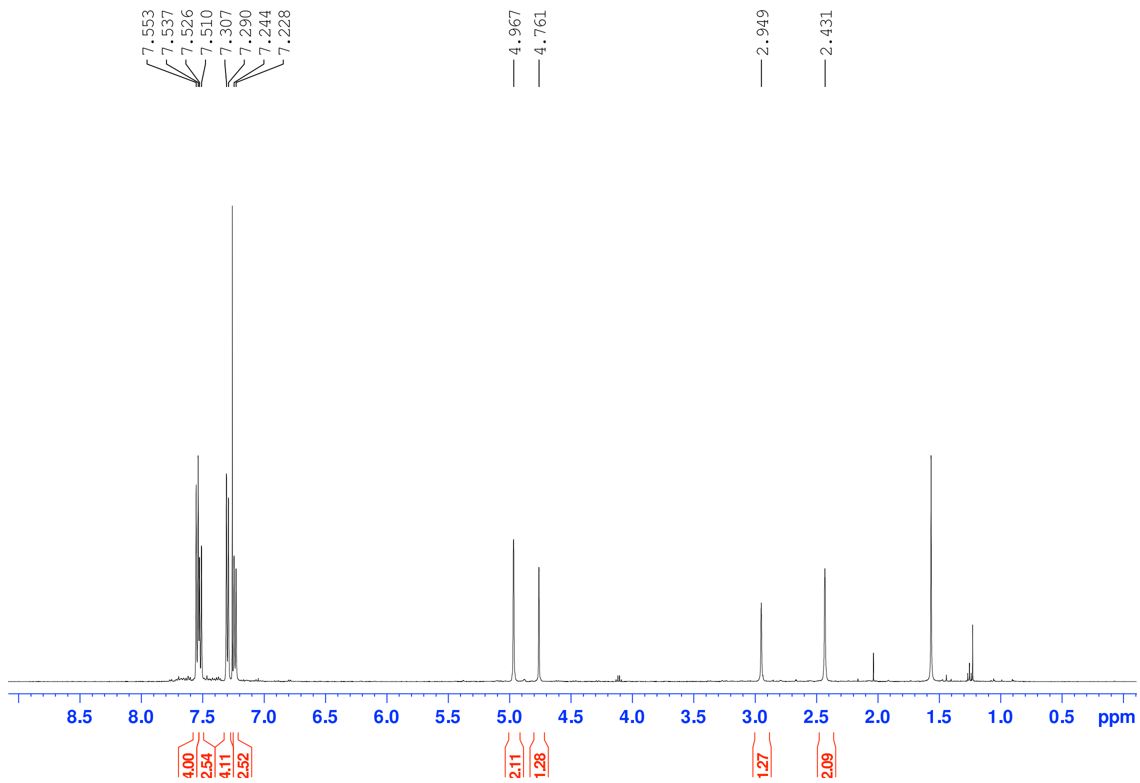

^{1}H NMR (500 MHz, $CDCl_3$) spectrum of compound *m*-**1b**

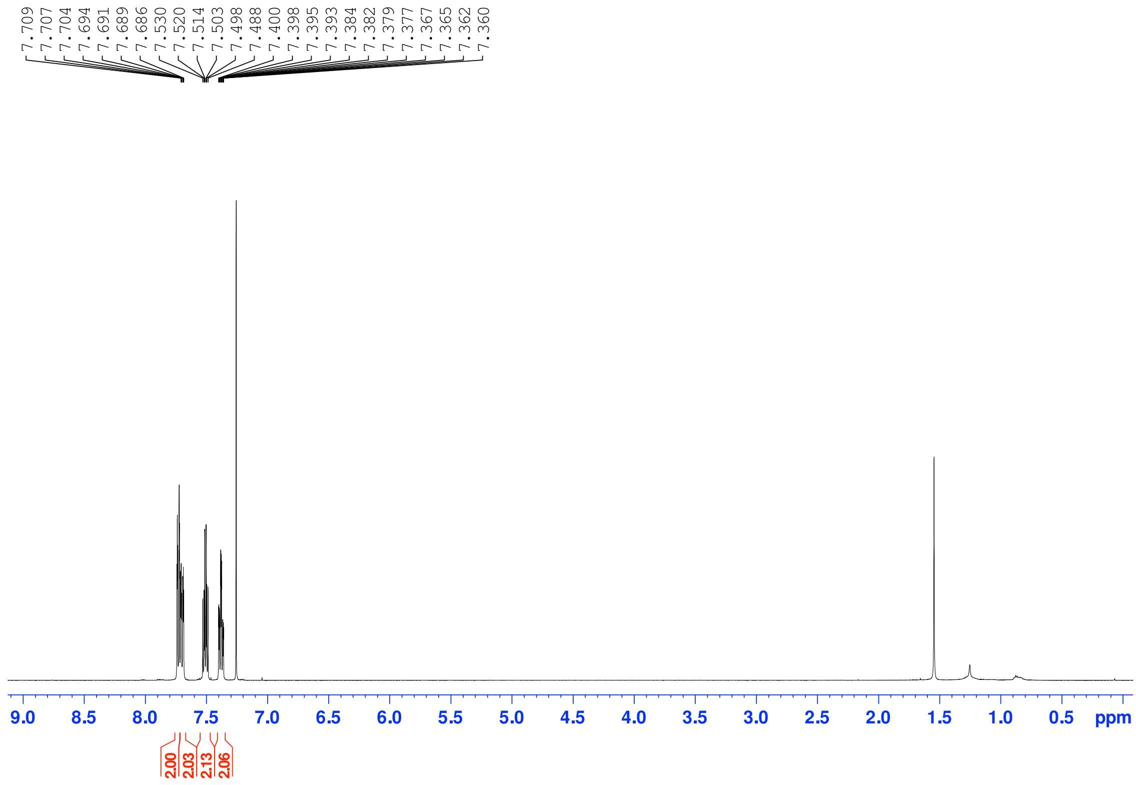

^1H NMR (500 MHz, $CDCl_3$) spectrum of compound *m*-**1c**

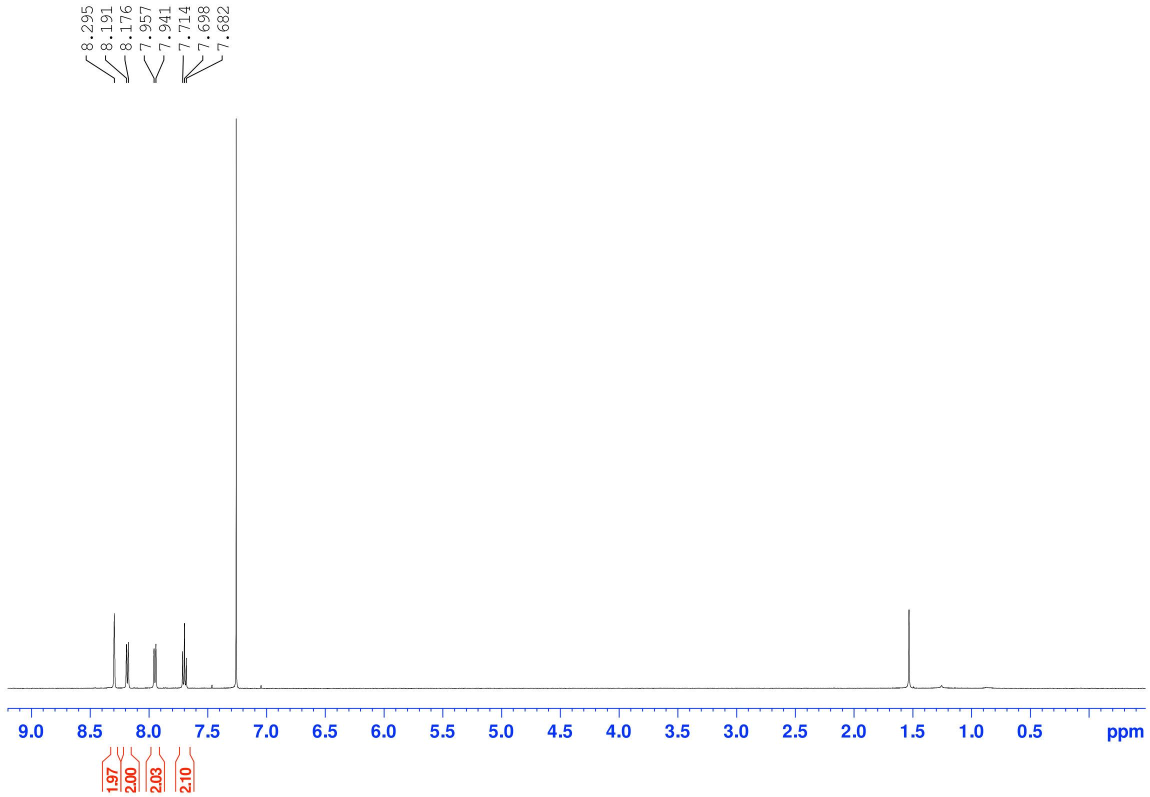

^1H NMR (500 MHz, $CDCl_3$) spectrum of compound *m*-**1d**

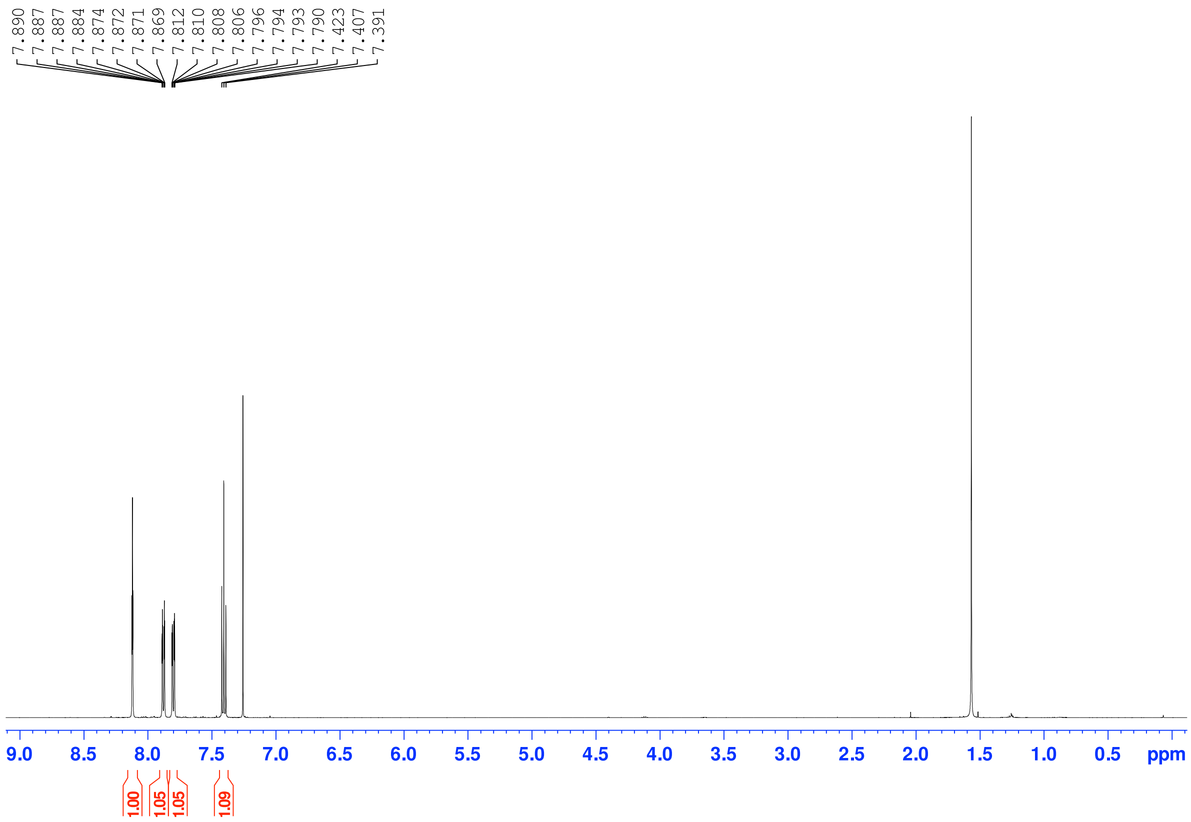

^{1}H NMR (500 MHz, $CDCl_3$) spectrum of compound *m*-**1e**

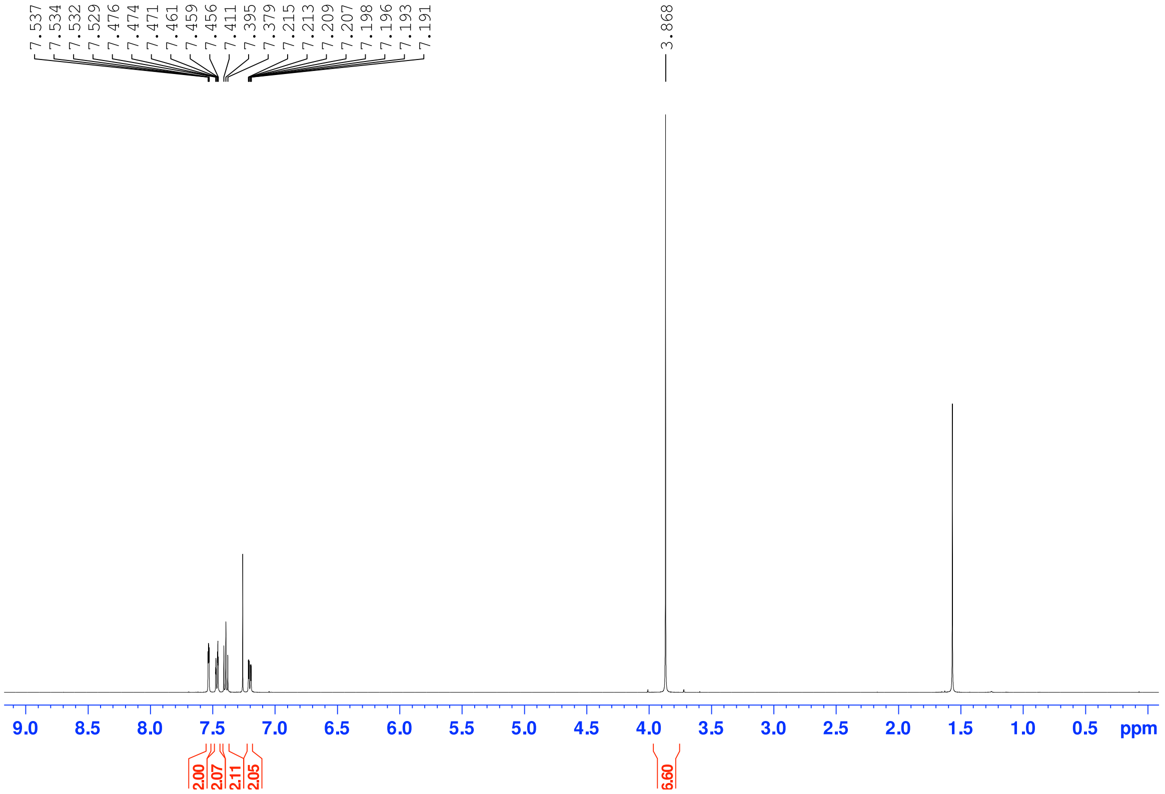

^{1}H NMR (500 MHz, $CDCl_3$) spectrum of compound *m*-**1f**

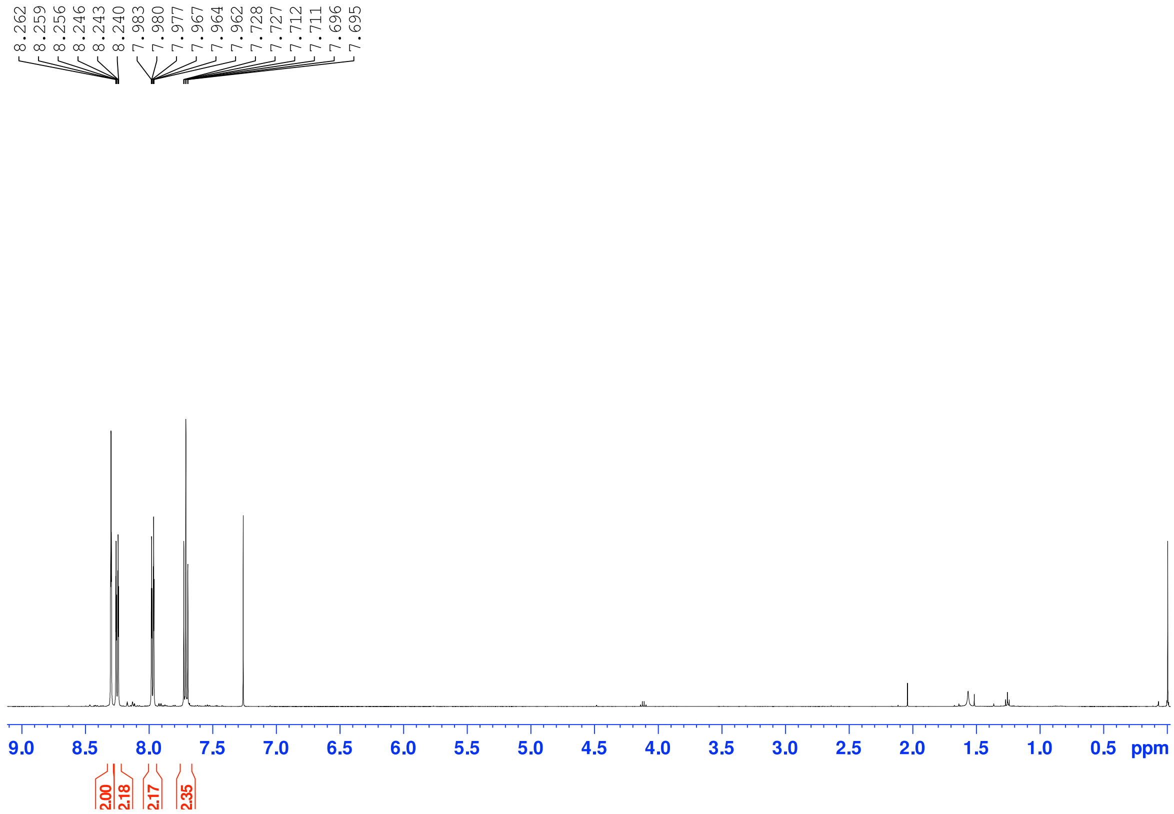

^{13}C NMR (126 MHz, $CDCl_3$) spectrum of compound *m*-**1f**

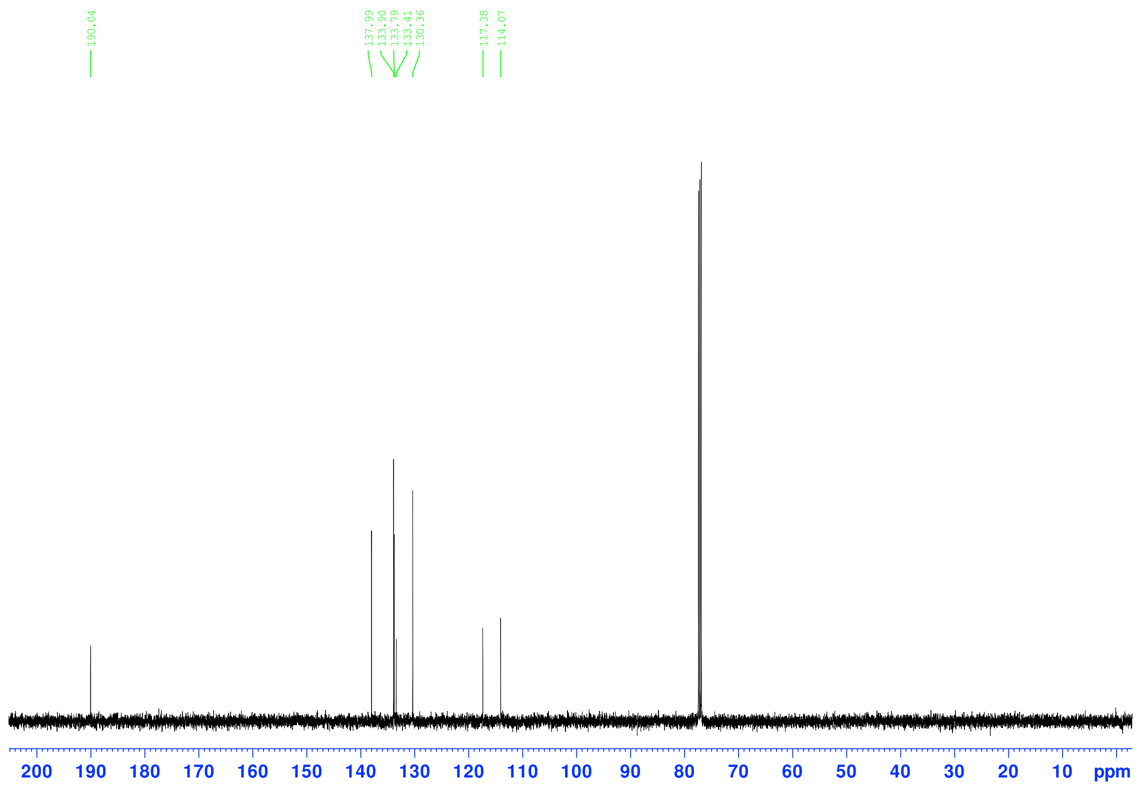

^{1}H NMR (500 MHz, $CDCl_3$) spectrum of compound *o*-**1b**

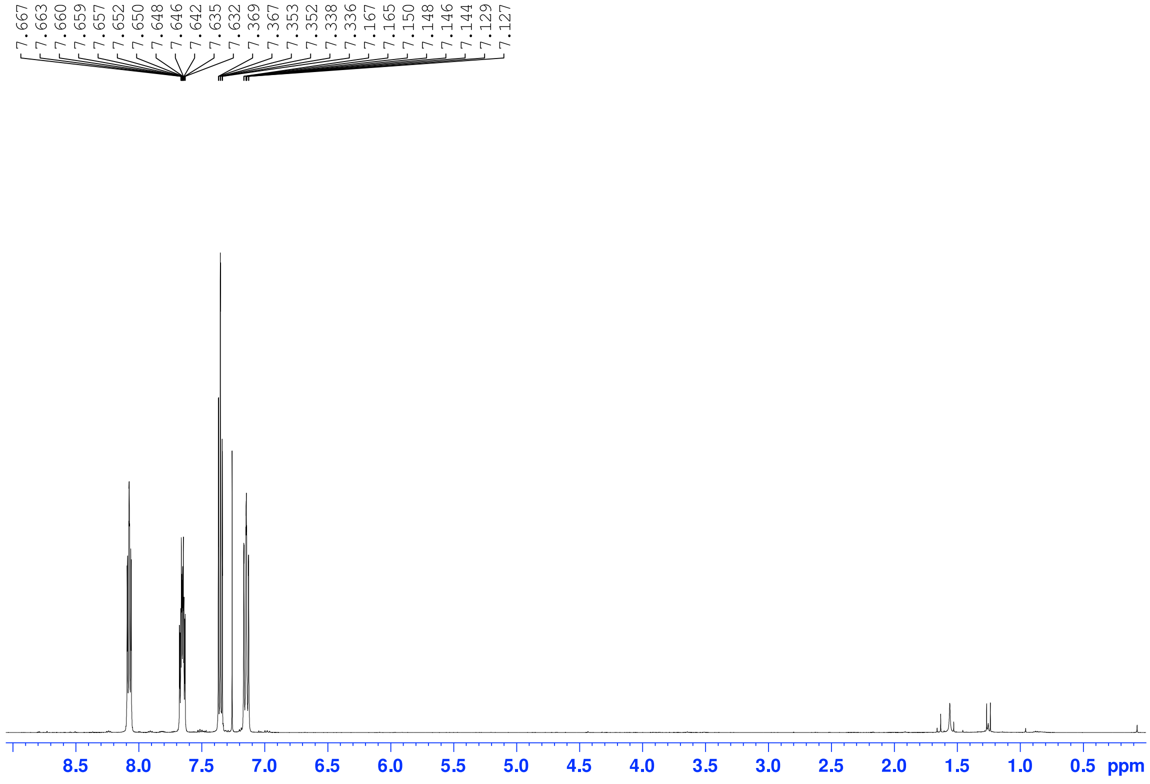

^{1}H NMR (500 MHz, $CDCl_3$) spectrum of compound *o*-**1c**

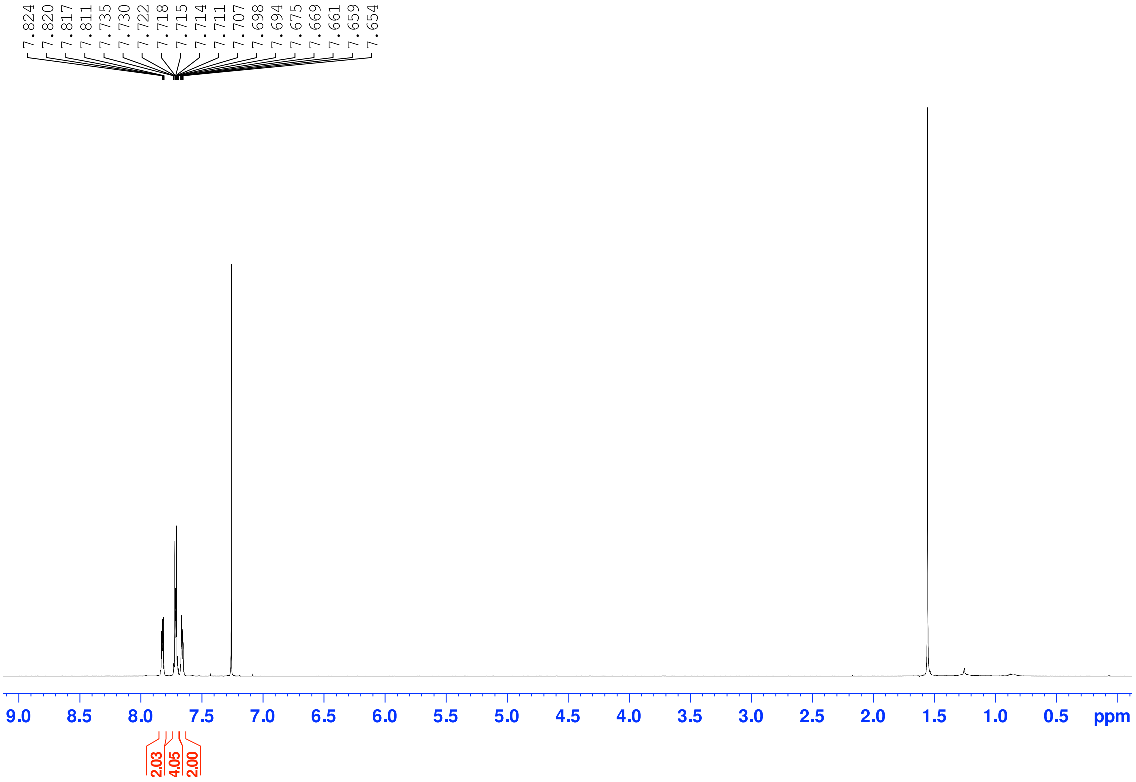

^{13}C NMR (126 MHz, $CDCl_3$) spectrum of compound *o*-**1c**

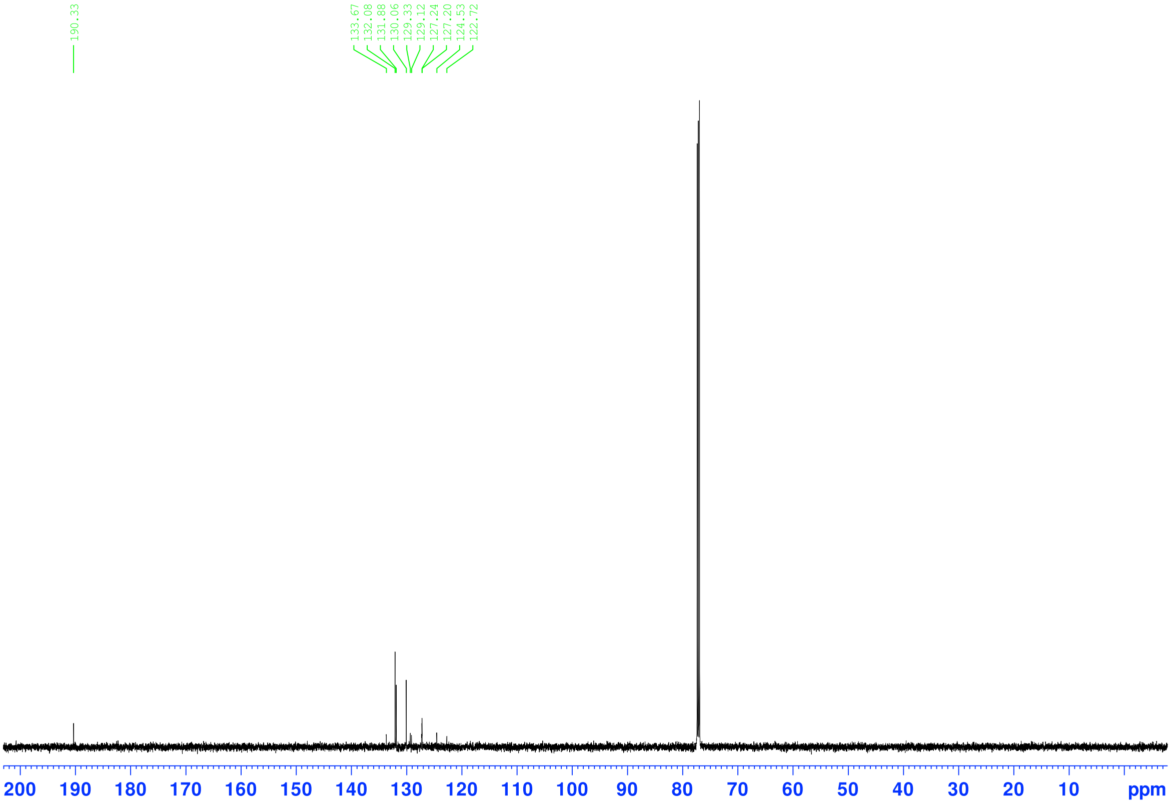

^{1}H NMR (500 MHz, $CDCl_3$) spectrum of compound *o*-**1d**

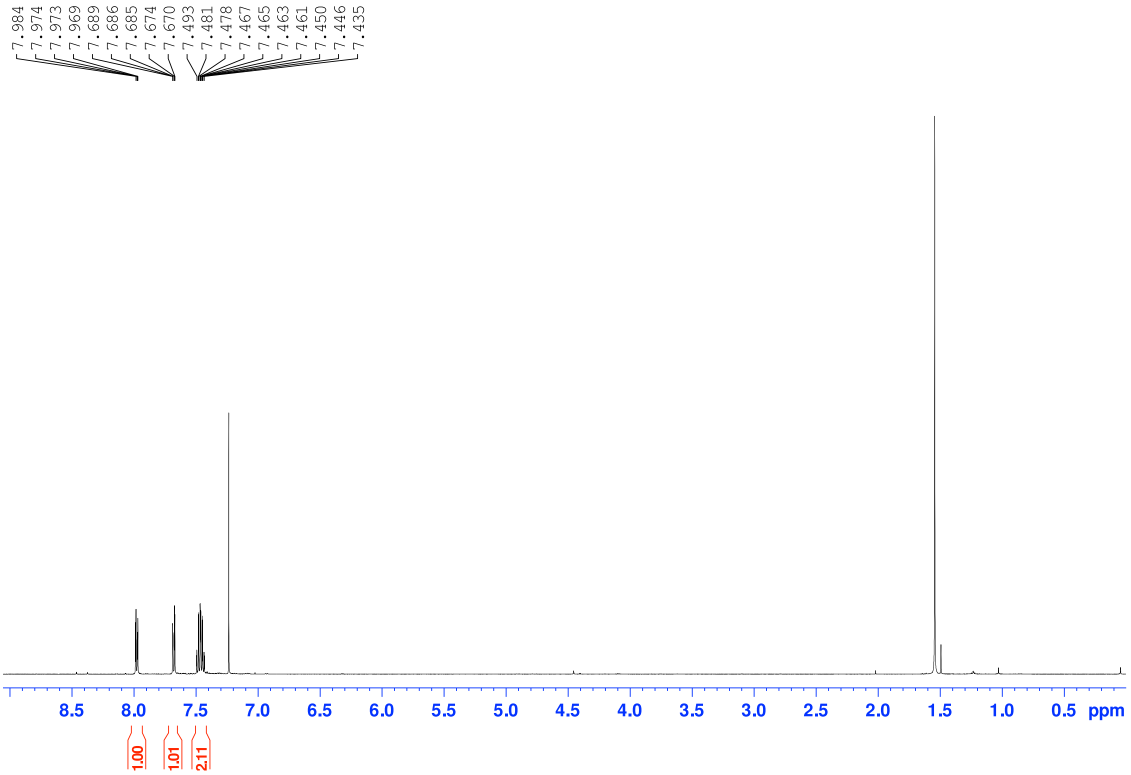

^{13}C NMR (126 MHz, $CDCl_3$) spectrum of compound *o*-**1d**

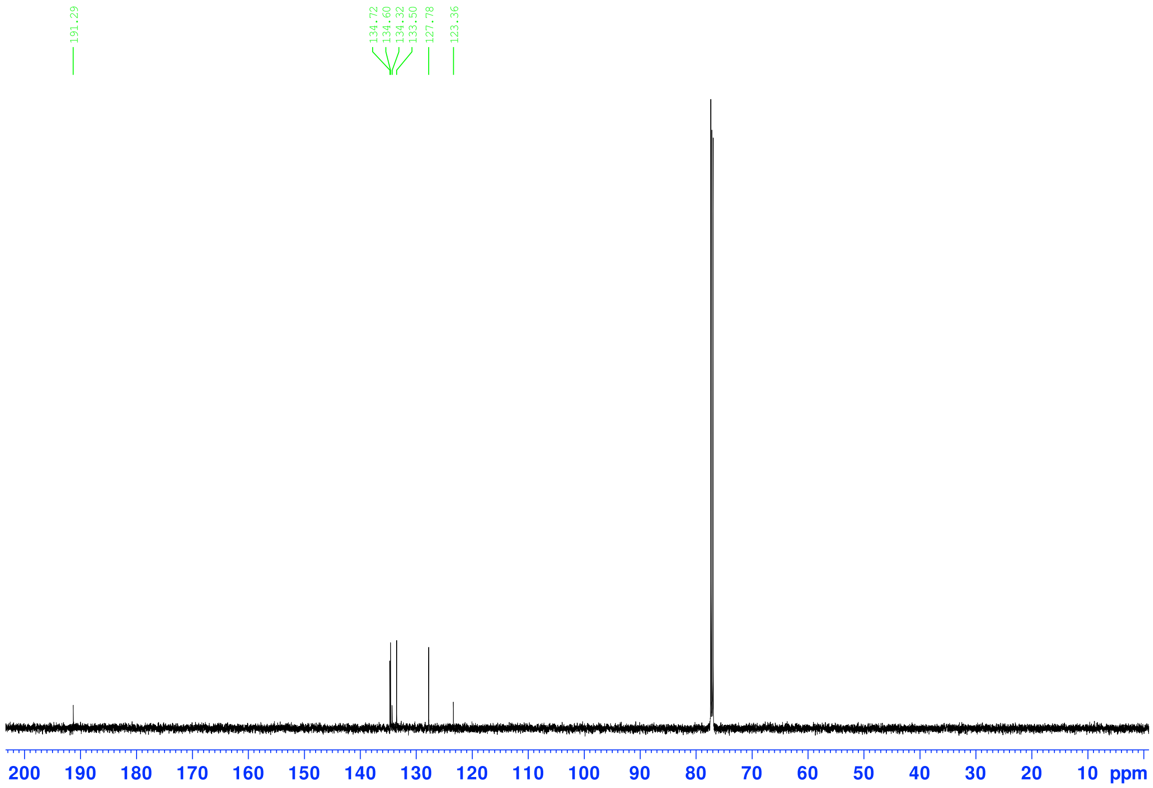

^{1}H NMR (500 MHz, $CDCl_3$) spectrum of compound *o*-**1e**

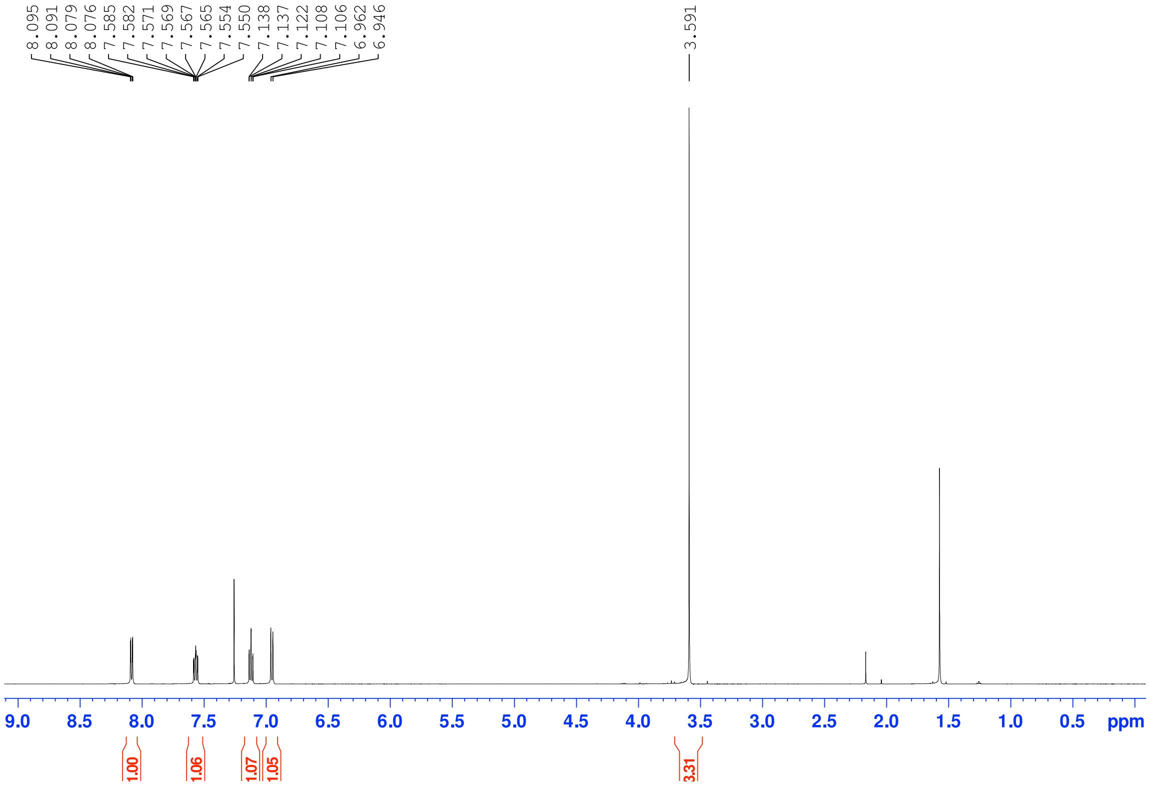

^{1}H NMR (500 MHz, $CDCl_3$) spectrum of compound *p*-**1c**

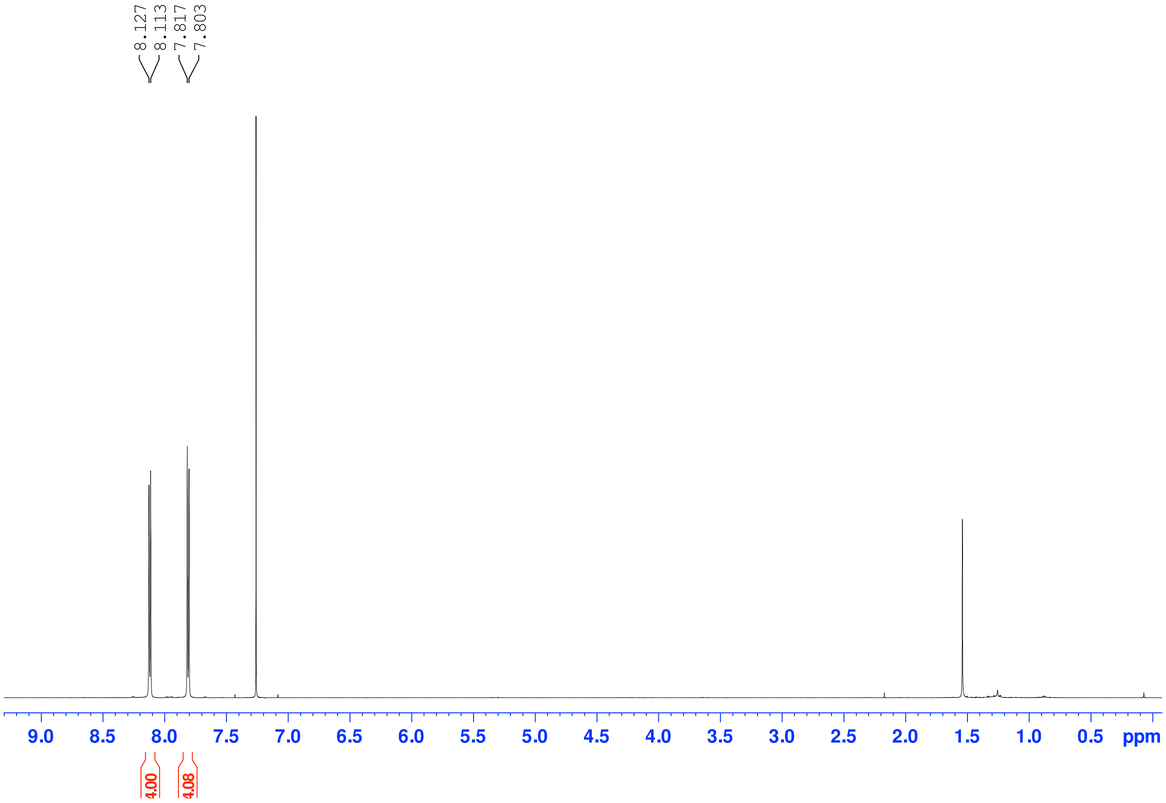

Supplement: Supplementary file 3 — Supplementary Data 1 [file 42004_2023_1038_MOESM3_ESM.pdf]
