# Supplementary material for: Synthesis and luminescence properties of substituted benzils
Source: Commun Chem. 2023 Nov 9;6:245. doi: 10.1038/s42004-023-01038-6 (PMC10636033; doi:10.1038/s42004-023-01038-6)
Supplement: Supplementary file 4 — Supplementary Data 2 [file 42004_2023_1038_MOESM4_ESM.pdf]

Supplementary Data 2

Synthesis and luminescence properties of substituted benzils

Masamichi Yasui,[1,2] Takashi Fujihara,[3]* Hiroyoshi Ohtsu,[4] Yuki Wada,[4] Terumasa Shimada,[4] Yiying Zhu,[4] Masaki Kawano,[4] Kengo Hanaya,[1] Takeshi Sugai[1] and Shuhei Higashibayashi[1]*

[1]Faculty of Pharmacy, Keio University, 1-5-30 Shibakoen, Minato-ku, Tokyo 105-8512, Japan

[2]Department of Chemistry, Graduate School of Science, Chiba University, 1-33 Yayoi, Inage, Chiba 263-8522, Japan.

[3]Comprehensive Analysis Center for Science, Saitama University, Shimo-okubo, Sakura-ku, Saitama-city, Saitama 338-8570, Japan

[4]Department of Chemistry, School of Science, Tokyo Institute of Technology, 2-12-1 Ookayama, Meguro-ku, Tokyo 152-8550, Japan

Theoretical Calculation

DFT and TD-DFT calculations were performed using the Gaussian 16 program package.[1] Calculations of spin-orbit couplings were done with the ORCA 5.0.3 program.[2] The structures were optimized at B3LYP/6-311+G(d,p) level of theory and the vibrational frequency analyses were conducted on the optimized structures. The given energies are zero-point corrected. TD-DFT calculation was conducted at CAM-B3LYP/6-311+G(d,p) level of theory.

***m*-1a (X = H)**

E = -689.935916 a.u.

O	1.57790	0.36379	1.40447
C	0.16835	1.87712	0.22332
C	1.10441	2.91958	0.30328
C	0.52014	0.57002	0.83862
C	-1.03896	2.08458	-0.45835
H	-1.77671	1.29473	-0.51357
C	-1.30318	3.31712	-1.04934
H	-2.23938	3.47415	-1.57230
C	0.83563	4.14805	-0.28469
H	1.56050	4.95122	-0.21767
C	-0.36918	4.34798	-0.96310
H	-0.57873	5.30765	-1.42237
O	-1.57790	-0.36379	1.40447
C	-0.16835	-1.87712	0.22332
C	-1.10441	-2.91958	0.30328
C	-0.52014	-0.57002	0.83862
C	1.03896	-2.08458	-0.45835
H	1.77671	-1.29473	-0.51357
C	1.30318	-3.31712	-1.04934
H	2.23938	-3.47415	-1.57230
C	-0.83563	-4.14805	-0.28469
H	-1.56050	-4.95122	-0.21767
C	0.36918	-4.34798	-0.96310
H	0.57873	-5.30765	-1.42237
H	-2.03332	-2.74298	0.83175
H	2.03332	2.74298	0.83175

***m*-1b (X = F)**

E = -888.487525 a.u.

C	-0.60442	0.47969	1.00814
C	-0.45770	1.84044	0.42092
C	0.71700	2.24778	-0.22642
C	-1.55466	2.71152	0.49273
C	0.75784	3.51498	-0.78162
H	1.59075	1.61356	-0.28580
C	-1.47809	3.97802	-0.07226
H	-2.45315	2.37598	0.99464
C	-0.31322	4.39302	-0.71969
H	-0.22808	5.37486	-1.16869
C	0.60442	-0.47969	1.00814
C	0.45770	-1.84044	0.42092
C	-0.71700	-2.24778	-0.22642
C	1.55466	-2.71152	0.49273
C	-0.75784	-3.51498	-0.78162
H	-1.59075	-1.61356	-0.28580
C	1.47809	-3.97802	-0.07226
H	2.45315	-2.37598	0.99464
C	0.31322	-4.39302	-0.71969
H	0.22808	-5.37486	-1.16869
O	1.62441	-0.10017	1.55034
O	-1.62441	0.10017	1.55034
H	-2.32521	4.65108	-0.01417
H	2.32521	-4.65108	-0.01417
F	1.89025	3.91327	-1.40662
F	-1.89025	-3.91327	-1.40662

***m*-1c (X = CF_3)**

E = -1364.221716 a.u.

C	0.35260	0.68655	-0.83940
C	-0.35312	1.86986	-0.27282
C	0.28918	3.11274	-0.36252
C	-1.59572	1.77756	0.36663
C	-0.30984	4.24250	0.17660
H	1.24784	3.17226	-0.86156
C	-2.18823	2.91592	0.90765
H	-2.10954	0.82759	0.42704
C	-1.55077	4.14816	0.81565
H	-2.01434	5.03540	1.22931

C	-0.35260	-0.68655	-0.83940
C	0.35312	-1.86986	-0.27282
C	-0.28918	-3.11274	-0.36252
C	1.59572	-1.77756	0.36663
C	0.30984	-4.24250	0.17660
H	-1.24784	-3.17226	-0.86156
C	2.18823	-2.91592	0.90765
H	2.10954	-0.82759	0.42704
C	1.55077	-4.14816	0.81565
H	2.01434	-5.03540	1.22931
O	-1.44581	-0.75991	-1.36591
O	1.44581	0.75991	-1.36591
H	-3.15254	2.84289	1.39559
H	3.15254	-2.84289	1.39559
C	0.39569	5.57314	0.12554
C	-0.39569	-5.57314	0.12554
F	-0.47100	6.59611	-0.05238
F	1.06087	5.82768	1.28107
F	1.30273	5.63654	-0.86946
F	-1.06087	-5.82768	1.28107
F	-1.30273	-5.63654	-0.86946
F	0.47100	-6.59611	-0.05238

***m*-1d (X = Br)**

E = -5837.038677 a.u.

C	-0.27280	-0.72216	1.53920
C	-1.61334	-0.99924	0.95188
C	-2.35255	-0.01029	0.28794
C	-2.11509	-2.30524	1.04164
C	-3.57863	-0.34594	-0.27378
H	-1.98934	1.00557	0.22436
C	-3.34419	-2.61754	0.47776
H	-1.52708	-3.05486	1.55604
C	-4.08611	-1.63904	-0.18662
H	-5.04438	-1.87950	-0.62895
C	0.27280	0.72216	1.53920
C	1.61334	0.99924	0.95188
C	2.35255	0.01029	0.28794
C	2.11509	2.30524	1.04164
C	3.57863	0.34594	-0.27378
H	1.98934	-1.00557	0.22436
C	3.34419	2.61754	0.47776
H	1.52708	3.05486	1.55604
C	4.08611	1.63904	-0.18662
H	5.04438	1.87950	-0.62895
O	-0.39796	1.57569	2.08587
O	0.39796	-1.57569	2.08587
H	-3.73566	-3.62567	0.54816
H	3.73566	3.62567	0.54816
Br	-4.59311	1.00188	-1.18328
Br	4.59311	-1.00188	-1.18328

***m*-1e (X = OMe)**

E = -918.983217 a.u.

C	0.66030	0.39909	-1.36591
C	0.70337	1.74021	-0.72192
C	1.89624	2.47878	-0.80210
C	-0.38831	2.24875	-0.01833
C	1.97330	3.71374	-0.18114
H	2.73385	2.06687	-1.34978
C	-0.30028	3.49596	0.61002
H	-1.32451	1.71003	0.04465
C	0.88365	4.23237	0.52764
H	0.97151	5.19982	1.00356
H	2.88755	4.29352	-0.23896
C	-0.66030	-0.39909	-1.36591
C	-0.70337	-1.74021	-0.72192
C	-1.89624	-2.47878	-0.80210
C	0.38831	-2.24875	-0.01833
C	-1.97330	-3.71374	-0.18114
H	-2.73385	-2.06687	-1.34978
C	0.30028	-3.49596	0.61002
H	1.32451	-1.71003	0.04465
C	-0.88365	-4.23237	0.52764
H	-2.88755	-4.29352	-0.23896
H	-0.97151	-5.19982	1.00356
O	-1.60733	0.08571	-1.95561
O	1.60733	-0.08571	-1.95561
O	1.42104	-3.90200	1.27081
O	-1.42104	3.90200	1.27081
C	-1.41313	5.17070	1.91277
H	-2.40016	5.28347	2.35760
H	-0.65338	5.21549	2.70064
H	-1.24456	5.98017	1.19441
C	1.41313	-5.17070	1.91277
H	2.40016	-5.28347	2.35760

H	0.65338	-5.21549	2.70064
H	1.24456	-5.98017	1.19441

***m*-1f (X = CN)**

E = -874.46538 a.u.

C	-0.40293	0.65842	0.75499
C	0.22095	1.89797	0.20950
C	-0.51038	3.08688	0.31167
C	1.47225	1.90551	-0.42197
C	0.00690	4.27258	-0.21190
H	-1.47682	3.07276	0.79941
C	1.98490	3.09073	-0.94447
H	2.05336	0.99606	-0.49221
C	1.26246	4.27288	-0.84318
H	1.65737	5.19734	-1.24622
C	0.40293	-0.65842	0.75499
C	-0.22095	-1.89797	0.20950
C	0.51038	-3.08688	0.31167
C	-1.47225	-1.90551	-0.42197
C	-0.00690	-4.27258	-0.21190
H	1.47682	-3.07276	0.79941
C	-1.98490	-3.09073	-0.94447
H	-2.05336	-0.99606	-0.49221
C	-1.26246	-4.27288	-0.84318
H	-1.65737	-5.19734	-1.24622
O	1.50517	-0.64683	1.26615
O	-1.50517	0.64683	1.26615
H	2.95407	3.09246	-1.42815
H	-2.95407	-3.09246	-1.42815
C	-0.74086	5.49010	-0.10998
N	-1.33968	6.47477	-0.03286
C	0.74086	-5.49010	-0.10998
N	1.33968	-6.47477	-0.03286

***o*-1b (X = F)**

E = -888.482332 a.u.

O	-1.51695	-0.37841	1.68700
C	-0.22684	-1.86689	0.36131
C	-0.94537	-3.00504	0.75772
C	-0.52508	-0.56390	1.01217
C	0.72401	-2.02290	-0.64795
C	0.98495	-3.24463	-1.24610
H	1.73153	-3.30515	-2.02781
C	-0.69972	-4.24166	0.17867
H	-1.25589	-5.11287	0.50269
C	0.26775	-4.36003	-0.82243
H	0.46328	-5.32330	-1.27919
O	1.51695	0.37841	1.68700
C	0.22684	1.86689	0.36131
C	0.94537	3.00504	0.75772
C	0.52508	0.56390	1.01217
C	-0.72401	2.02290	-0.64795
C	-0.98495	3.24463	-1.24610
H	-1.73153	3.30515	-2.02781
C	0.69972	4.24166	0.17867
H	1.25589	5.11287	0.50269
C	-0.26775	4.36003	-0.82243
H	-0.46328	5.32330	-1.27919
F	1.40708	-0.94039	-1.08730
F	-1.40708	0.94039	-1.08730
H	-1.69172	-2.88522	1.53365
H	1.69172	2.88522	1.53365

***o*-1c (X = CF_3)**

E = -1364.200434 a.u.

F	-3.41050	1.91653	-1.14599
F	-2.34228	1.94632	0.75103
F	-4.48726	1.62100	0.70888
O	-0.55466	0.92922	-1.35629
C	-1.83493	-0.72123	-0.20530
C	-3.11577	-0.16795	-0.02662
C	-0.61684	0.12064	-0.46192
C	-3.33550	1.32594	0.06382
C	-1.69388	-2.11173	-0.23416
H	-0.70895	-2.54555	-0.34944
C	-2.80633	-2.94159	-0.13140
H	-2.67920	-4.01698	-0.16959
C	-4.22285	-1.00572	0.09667
H	-5.20243	-0.57436	0.25441
C	-4.07164	-2.38863	0.03356
H	-4.94125	-3.02821	0.12662
F	3.41050	-1.91653	1.14599
F	2.34228	-1.94632	-0.75103
F	4.48726	-1.62100	-0.70888
O	0.55466	-0.92922	1.35629
C	1.83493	0.72123	0.20530

C	3.11577	0.16795	0.02662
C	0.61684	-0.12064	0.46192
C	3.33550	-1.32594	-0.06382
C	1.69388	2.11173	0.23416
H	0.70895	2.54555	0.34944
C	2.80633	2.94159	0.13140
H	2.67920	4.01698	0.16959
C	4.22285	1.00572	-0.09667
H	5.20243	0.57436	-0.25441
C	4.07164	2.38863	-0.03356
H	4.94125	3.02821	-0.12662

***o*-1d (X = Br)**

E = -5837.023194 a.u.

O	1.55627	0.14355	1.94893
C	0.43446	1.90130	0.81280
C	1.25962	2.85511	1.43283
C	0.58892	0.49682	1.30574
C	-0.43671	2.34588	-0.19274
C	-0.49090	3.69090	-0.55219
H	-1.16633	4.00833	-1.33570
C	1.20786	4.19643	1.08713
H	1.84792	4.91188	1.58933
C	0.32523	4.61513	0.09227
H	0.27093	5.66033	-0.19015
O	-1.55627	-0.14355	1.94893
C	-0.43446	-1.90130	0.81280
C	-1.25962	-2.85511	1.43283
C	-0.58892	-0.49682	1.30574
C	0.43671	-2.34588	-0.19274
C	0.49090	-3.69090	-0.55219
H	1.16633	-4.00833	-1.33570
C	-1.20786	-4.19643	1.08713
H	-1.84792	-4.91188	1.58933
C	-0.32523	-4.61513	0.09227
H	-0.27093	-5.66033	-0.19015
Br	1.56212	-1.15707	-1.19345
Br	-1.56212	1.15707	-1.19345
H	1.93744	2.50312	2.20074
H	-1.93744	-2.50312	2.20074

***o*-1e (X = OMe)**

E = -918.980555 a.u.

O	0.31676	-1.51536	-1.95269
C	1.84877	-0.33027	-0.57697
C	2.04151	0.62983	0.43884
C	0.53854	-0.55043	-1.24536
C	2.93452	-1.11119	-0.98675
H	2.75841	-1.84505	-1.76424
C	4.19599	-0.94157	-0.43089
H	5.02953	-1.54593	-0.76756
C	3.31186	0.80707	0.99700
H	3.47430	1.54260	1.77291
C	4.37731	0.02432	0.55786
H	5.35689	0.17353	0.99869
O	-0.31676	1.51536	-1.95269
C	-1.84877	0.33027	-0.57697
C	-2.04151	-0.62983	0.43884
C	-0.53854	0.55043	-1.24536
C	-2.93452	1.11119	-0.98675
H	-2.75841	1.84505	-1.76424
C	-4.19599	0.94157	-0.43089
H	-5.02953	1.54593	-0.76756
C	-3.31186	-0.80707	0.99700
H	-3.47430	-1.54260	1.77291
C	-4.37731	-0.02432	0.55786
H	-5.35689	-0.17353	0.99869
O	0.94306	1.31891	0.83751
O	-0.94306	-1.31891	0.83751
C	1.08945	2.42530	1.72137
H	1.78044	3.16818	1.31165
H	0.09692	2.86281	1.81074
H	1.43430	2.10160	2.70877
C	-1.08945	-2.42530	1.72137
H	-0.09692	-2.86281	1.81074
H	-1.43430	-2.10160	2.70877
H	-1.78044	-3.16818	1.31165

1. Gaussian 16, Revision C.02, Frisch, M. J. *et al.* Gaussian, Inc., Wallingford CT (2019).
2. Neese, F., Wennmohs, F., Becker, U. & Riplinger, C. The ORCA quantum chemistry program package. *J. Chem. Phys.* **152**, 224108 (2020).
